# Antiviral Activity of Essential Oil from *Populus balsamifera* L. Buds and Its Major Compounds Against Betacoronavirus HCoV-OC43 Using a Sensitive Cytoprotection Assay

**DOI:** 10.3390/molecules31091496

**Published:** 2026-04-30

**Authors:** Laurie Girard, Héloïse Côté, Andre Pichette, Josianne Savard, Lionel Ripoll, Jean Legault

**Affiliations:** 1 Laboratoire d’Analyse et de Séparation des Essences Végétales, Université du Québec à Chicoutimi, Chicoutimi, QC G7H 2B1, Canada; lgirard27@etu.uqac.ca (L.G.); hcote@etu.uqac.ca (H.C.); andre_pichette@uqac.ca (A.P.); lripoll@uqac.ca (L.R.); 2 Centre de Recherche sur la Boréalie (CREB), Département des Sciences Fondamentales, Université du Québec à Chicoutimi, Chicoutimi, QC G7H 2B1, Canada

**Keywords:** *Populus balsamifera* L., buds, essential oil, cytoprotection, antiviral activity, sensitive antiviral assay, α-bisabolol, nerolidol, betacoronavirus, HCoV-OC43

## Abstract

Plant-derived products, particularly essential oils, represent a promising source of antiviral scaffolds. Although *Populus balsamifera* L. has been traditionally used to manage respiratory ailments and infections, the antiviral potential of its bud essential oil remains unexplored. In this study, we evaluated the *in vitro* antiviral cytoprotective activity of *P. balsamifera* bud essential oil and its major constituents against HCoV-OC43, a human betacoronavirus associated with seasonal respiratory infections, using a cell-based cytoprotection assay. The assay reliably detected the activity of reference antivirals, including chloroquine (EC_50_ = 0.11 ± 0.01 µg/mL), molnupiravir, and fluvoxamine, supporting its suitability for antiviral screening. Under these conditions, *P. balsamifera* bud essential oil exhibited strong cytoprotective activity, with an EC_50_ of 3.3 ± 0.5 µg/mL. Chemical analysis revealed a sesquiterpene-rich composition. Two major constituents, α-bisabolol and nerolidol, also showed marked cytoprotection, with EC_50_ values of 2.7 ± 0.3 µg/mL and 2.6 ± 0.4 µg/mL, respectively, supporting their contribution to the overall activity of the oil. To our knowledge, this study provides the first experimental evidence of antiviral cytoprotective activity of *P. balsamifera* bud essential oil against a human coronavirus and identifies α-bisabolol and nerolidol as active compounds in this model.

## 1. Introduction

Human coronaviruses contribute substantially to seasonal respiratory infections and can cause severe disease in vulnerable populations [1]. Among the seven human coronaviruses identified so far, namely HCoV-229E, HCoV-HKU1, HCoV-NL63, HCoV-OC43, SARS-CoV, SARS-CoV-2 and MERS-CoV, commons HCoVs contribute to 15–30% of cases of common colds [1,2]. While more than 100 antivirals drugs and combinations have been approved for clinical use, most are specific to a limited number of infectious viral diseases: cytomegalovirus (CMV), hepatitis B virus (HBV), hepatitis C virus (HCV), herpes simplex virus (HSV), human immunodeficiency virus (HIV), influenza virus, respiratory syncytial virus (RSV), varicella-zoster virus (VZV) and variola virus (human smallpox) [3]. However, several other viruses such as coronaviruses still lack effective and standardized therapeutic options [4]. This is partly due to scientific, technical and economic limitations associated with the slow and challenging process of antiviral drug development [3,5,6]. Accordingly, antiviral activity is commonly evaluated using *in vitro* assays, including cytopathic effect-based methods (plaque reduction and virus yield reduction assays) [7,8], detection-based methods (ELISA and PCR) [9], and engineered systems (replicon and reporter gene assays) [10], which provide useful and high-throughput platforms for antiviral screening. These approaches remain nonetheless limited by variability, labor-intensive and time-consuming procedures, high cost, lack of standardization, and limited applicability across different virus types [8,11,12,13].

In this context, the exploration of alternative approaches is required. In particular the development of novel antiviral strategies remains a priority, especially for coronaviruses, for which therapeutic options are still limited despite intensified research since the COVID-19 pandemic [14,15]. Essential oils are widely recognized for their diverse bioactivities [16,17,18], and several have demonstrated *in vitro* antiviral effects against influenza viruses, herpesviruses, and human coronaviruses, including HCoV-229E and HCoV-OC43, as well as SARS-CoV and SARS-CoV-2 [19,20,21]. Interest in essential oils as sources of antiviral leads has consequently increased [20,22,23]. Nevertheless, essential oils from boreal plants remain comparatively understudied with respect to antiviral potential [24]. *Populus balsamifera* L. (balsam poplar; Salicaceae) is widely distributed in Canada and the northern United States [25]. Ethnobotanical reports describe the use of balsam poplar preparations to treat wounds, headaches, respiratory complaints, influenza-like illness, and colds [26,27,28,29,30]. Traditional Canadian preparations methods of this plant included decoction, poultice, infusion, bath preparation, inhalation (steam) and tea [30,31]. Pharmacological studies of *P. balsamifera* buds and extracts have reported anti-psoriatic, anti-inflammatory, antioxidant, antibacterial, anti-obesity, and antidiabetic properties [27,32]. However, antiviral activity against betacoronaviruses has not been documented for this species.

In this study, we developed a sensitive cytoprotection assay to evaluate antiviral activity against HCoV-OC43, a human betacoronavirus used for antiviral screening under biosafety level 2 (BSL-2) conditions [2]. We first selected the most sensitive host cell line and validated the assay using reference controls. The validated platform was then used to assess the antiviral cytoprotective activity of *P. balsamifera* bud essential oil, followed by GC-MS/GC-FID characterization and evaluation of selected major constituents. We hypothesized that both the essential oil and its major constituents may exert antiviral cytoprotective effects against HCoV-OC43. This study addresses the limited knowledge on the antiviral potential of boreal plant essential oils, particularly *P. balsamifera*, against betacoronaviruses.

## 2. Materials and Methods

### 2.1. Compounds

Chloroquine phosphate (PHR1258) and molnupiravir (SML2873, EIDD-2801, ≥98%) were purchased from MilliporeSigma (Burlington, MA, USA). Fluvoxamine maleate (F0858, >98%) and hydroxychloroquine sulfate (H1306, >98%) were supplied by Tokyo Chemical Industry (Tokyo, Japan). The essential oil from *P. balsamifera* buds (Lot: B-POBA-CAN-05-E) was obtained from Aliksir (Grondines, QC, Canada). The main compounds of the essential oil, α-bisabolol (≥93%) and nerolidol (98%), were purchased respectively from Fluka (14462, Buchs, Switzerland) and Sigma-Aldrich (960-5, St. Louis, MO, USA).

### 2.2. Cell Lines

The human lung fibroblasts MRC-5 (CCL-171), IMR-90 (CCL-186), and WI-38 (CCL-75) and human colorectal adenocarcinoma HCT-8 (CCL-244) were obtained from the American Type Culture Collection (ATCC, Manassas, VA, USA). MRC-5 cells are widely used in virology research and vaccine production [33,34]. HCT-8 cells were used for viral replication of HCoV-OC43 (VR-1558), as recommended by the ATCC. All cell lines were grown in a humidified atmosphere at 37 °C and 5% CO_2_. The three human lung cell lines were maintained in Minimum Essential Medium Eagle (MEM, 220-005-XK, Wisent Bioproducts Inc., Saint-Jean-Baptiste, QC, Canada) supplemented with 10% fetal bovine serum (FBS), 1% penicillin–streptomycin (Thermo Fisher Scientific Inc., Waltham, MA, USA), and 0.001% sodium pyruvate (SH3023901, Thermo Fisher Scientific Inc., USA). The culture medium used for the growth of HCT-8 cells was RPMI-1640 (250-010-XK, Wisent Bioproducts Inc., Saint-Jean-Baptiste, QC, Canada) supplemented with 10% FBS, 1% penicillin–streptomycin, and 0.001% sodium pyruvate. No human or animal subjects were used in this study.

### 2.3. Virus Amplification and Purification

Virus amplification was performed as recommended by the ATCC. Briefly, a monolayer of HCT-8 cells was incubated for one day in a T25 flask to achieve a minimum of 80% confluence. A total of 1 mL of virus was added to the monolayer and allowed to adsorb for 1 h at 37 °C and 5% CO_2_. Subsequently, 5 mL of RPMI-1640 medium containing 2% FBS was added, and the cells were incubated for four to six days under the same conditions until the cytopathic effect reached 80%. The supernatant and HCT-8 cells were centrifuged at 3000 rpm (Beckman Coulter, Brea, CA, USA, rotor: S4180) for 30 min at 4 °C. The pellet was resuspended in 5 mL of Hanks’ Balanced Salt Solution (HBSS) medium and transferred to a 50 mL tube to undergo three freeze–thaw cycles. The samples were then centrifuged for 20 min under the same conditions. The supernatant was subsequently centrifuged at 15,900 rpm (Beckman Coulter, rotor: JA-20) for 160 min at 4 °C under vacuum. The supernatant was discarded, and the pellet was resuspended in 5 mL of HBSS, aliquoted into cryotubes, and frozen at −80 °C.

### 2.4. Screening for Host–Cell Selection

A cell line screening was conducted to determine the most suitable cell line for the antiviral assay. Four different cell lines—HCT-8, WI-38, IMR-90, and MRC-5—were seeded into 96-well microplates at a density of 1 × 10^4^ cells/well. The cells were allowed to attach by incubating for 24 h at 37 °C and 5% CO_2_ until reaching 90% confluence, so as to minimize active cell proliferation during the assay. Under these conditions, changes in cell viability are unlikely to reflect cytostatic effects related to inhibition of cell growth. Cells at 90% confluence were then infected with a growing gradient of HCoV-OC43 for three days at 37 °C and 5% CO_2_. Viral infection induced a pronounced cytopathic effect, characterized by cell detachment and lysis, as routinely observed by microscopic examination during the assay. For viral infection, the medium for each cell line was supplemented with 2% FBS. The MRC-5 cell line, which exhibited the best dose–response curve, was selected to evaluate the antiviral activity of the positive controls, essential oil, and compounds.

### 2.5. Determination of Viral Titration Using a Cytotoxic Assay

The appropriate viral inoculum for the cytoprotection assay was established by titrating HCoV-OC43 on MRC-5 monolayers and selecting conditions that yielded a broad, quantifiable window of virus-induced cytotoxicity. Briefly, MRC-5 cells were seeded in 96-well plates at 1 × 10^4^ cells/well in 100 µL of complete MEM and incubated for 24 h at 37 °C and 5% CO_2_ to allow monolayer formation. A purified HCoV-OC43 stock (defined as the undiluted inoculum; relative viral inoculum = 1) was then serially diluted 1:2 in MEM supplemented with 2% FBS to generate seven viral concentrations. Each dilution (100 µL) was added to the appropriate wells, and plates were incubated for 72 h at 37 °C and 5% CO_2_. Virus-induced cytotoxicity was quantified using a resazurin reduction assay [35]. After incubation, wells were washed once with phosphate-buffered saline (PBS) and incubated with 100 µL of resazurin solution (50 µg/mL) for 1 h. Fluorescence was measured using a Fluoroskan Ascent FL plate reader (Labsystems, Milford, MA, USA) with excitation at 530 nm and emission at 590 nm. Cell viability was calculated relative to uninfected controls based on the fluorescence signal. Under these conditions, viral titration established an operational assay window spanning approximately 20% to 90% virus-induced mortality, corresponding to a relative viral concentration of ~0.015 (from the serial dilution series) and the undiluted inoculum [1], respectively.

### 2.6. Evaluation of Maximum Tolerated Concentration

The maximum tolerated concentration (MTC) of positive controls (chloroquine, hydroxychloroquine, molnupiravir, fluvoxamine), *P. balsamifera* bud essential oil, α-bisabolol, and nerolidol is the respective concentration causing less than 20% mortality of the MRC-5 cells. First, MRC-5 cells were seeded into 96-well microplates in 100 µL MEM complete medium with 10% FBS at a density of 1 × 10^4^ cells/well and incubated overnight at 37 °C and 5% CO_2_. The following day, cells were treated with increasing concentrations of positive controls, *P. balsamifera* bud essential oil, or compounds dissolved in their respective solvents. DMSO was used for molnupiravir, *P. balsamifera* essential oil, α-bisabolol, and nerolidol; water was used to dissolve chloroquine phosphate, fluvoxamine maleate, and hydroxychloroquine sulfate. To avoid solvent toxicity, we ensured that the final concentration of DMSO in the culture medium was less than 0.25% (*v*/*v*). We assessed cytotoxicity at 72 h after incubation using the resazurin reduction test [35].

### 2.7. Evaluation of Antiviral Activity

A cytoprotective antiviral assay was developed to enable rapid screening of antiviral activity under BSL-2 conditions. This assay is based on the quantification of virus-induced cytopathic effects on host cell viability using a resazurin reduction readout. In contrast to conventional antiviral assays that directly quantify viral replication, this approach measures the ability of compounds to protect host cells from virus-induced cell death, providing an indirect but sensitive indicator of antiviral activity. MRC-5 cells were seeded in 96-well plates at a density of 1 × 10^4^ cells/well in MEM complete medium supplemented with 10% FBS and incubated overnight at 37 °C and 5% CO_2_. The following day, the medium was replaced with 50 µL of MEM complete medium containing 2% FBS and the tested compounds. Concentrations were derived from the maximum tolerated concentration (MTC) determined in cytotoxicity assays and tested using serial 1:2 dilutions. The concentration ranges were as follows: chloroquine (0.04–1.25 µg/mL), hydroxychloroquine (0.05–1.6 µg/mL), molnupiravir (0.4–12.5 µg/mL), fluvoxamine (0.4–12.5 µg/mL), *P. balsamifera* bud essential oil (0.2–14.4 µg/mL), α-bisabolol (0.2–14.4 µg/mL), and nerolidol (0.2–14.4 µg/mL). Cells were then infected with HCoV-OC43 using a serial dilution scheme. After 72 h of incubation at 37 °C and 5% CO_2_, cell viability was assessed using a resazurin reduction assay [35]. Antiviral activity was defined as the cytoprotection provided by the tested compounds against virus-induced cell death. Cytoprotective effects were quantified using an area under the curve (AUC)-based approach. The area under the curve of the virus-only condition (AUC_v_) and of treated conditions (AUC_c_) were calculated, and AUC_100_, corresponding to complete protection, was estimated. Antiviral protection (%) was calculated as follows:

Antiviral protection (%) = (AUC_c_ − AUC_v_)/(AUC_100_ − AUC_v_) × 100

The half-maximal effective concentration (EC_50_) was defined as the concentration providing 50% cytoprotection. EC_50_ values were determined by fitting dose–response curves using a non-linear sigmoidal regression model (four-parameter logistic model). Chloroquine was used as a positive control to benchmark assay performance. All experiments were performed in at least three independent biological replicates (*n* ≥ 3).

### 2.8. Chromatographic Analysis

Gas chromatography–mass spectrometry (GC-MS) and gas chromatography with flame ionization detection (GC-FID) were used to identify and quantify the components of the essential oil. Chromatographic analyses were conducted using an Agilent 6890 GC (Agilent Technologies, Santa Clara, CA, USA) equipped with a non-polar DB-5 column and a flame ionization detector (FID). The oils were injected in a non-dried and undiluted state (1 µL injection volume, split 1:235). The temperature program began at 40 °C for 3 min, increased at 3 °C·min^−1^ to 210 °C, and was then held at 210 °C for 13 min. The essential oil samples were also injected into an Agilent 7890A GC system (Agilent Technologies, Santa Clara, CA, USA) coupled to an Agilent 5975C EI MSD with Triple-Axis Detector equipped with a DB-5MS column using the same temperature program and a split of 1:235 (Agilent Technologies, Santa Clara, CA, USA). Compounds were identified from their retention indexes as calculated from even-numbered C7 to C40 alkane standards and/or from MS databases (e.g., NIST library) [36,37,38]. Quantification was based on the FID response in the DB-5 column, without correction factors. Only components with an abundance greater than 0.5% were analyzed.

### 2.9. Statistical Analyses

Statistical analyses were performed using SigmaStat software (version 3.5, Systat Software Inc., San Jose, CA, USA). Cell survival percentages were analyzed using a two-way ANOVA followed by a Holm–Sidak multiple comparison test. A two-way ANOVA followed by a Holm–Sidak multiple comparison test was also performed on the produced AUC. We ran a one-way ANOVA followed by a Holm–Sidak multiple comparison test on the AUC to compare the compounds with the viral curve. Differences were considered statistically significant at *p* < 0.05.

## 3. Results and Discussion

### 3.1. Host Cell Selection and Viral Titration to Define the Assay Window

**Figure 1:**
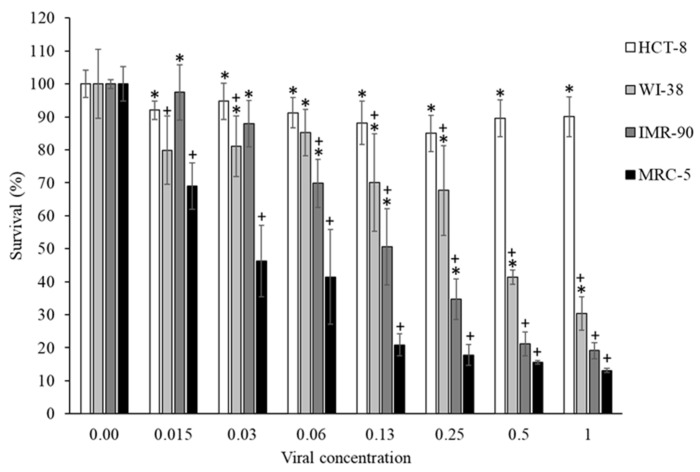
Cytotoxicity induced by HCoV-OC43 infection in human colon and lung cell lines. Viral concentrations correspond to a 1:2 serial dilution, where 1 represents the undiluted viral inoculum. For all data, *n* ≥ 3 (biological replicates). + Significantly different from uninfected cells (*p* < 0.05). * Significantly different from MRC-5 cells (*p* < 0.05). Data are representative of three separate experimental runs.

To evaluate antiviral cytoprotective activity under accessible laboratory conditions, HCoV-OC43 was selected as a model human coronavirus that can be handled at biosafety level 2 (BSL-2). Among the four tested cell lines, the three human lung fibroblast cell lines (WI-38, IMR-90, and MRC-5) exhibited greater virus-induced cell death than the human colon cell line HCT-8 (Figure 1), consistent with the respiratory tropism of HCoV-OC43 [39,40]. Among them, MRC-5 cells showed the highest susceptibility to infection and were therefore selected for subsequent experiments. This choice is further supported by their frequent use in HCoV-OC43 infection studies [41,42,43]. Viral titration in MRC-5 cells established a broad and quantifiable assay window, with virus-induced mortality ranging from approximately 20% to 90% across the dilution series. This dynamic range provided suitable conditions for detecting and quantifying cytoprotective effects of tested extracts and compounds.

### 3.2. Assay Validation Using Reference Controls

The antiviral cytoprotection assay quantifies the ability of extracts or compounds to protect MRC-5 cells from HCoV-OC43-induced cytotoxicity. This protective effect may reflect inhibition of viral entry and/or replication, or modulation of host stress and survival pathways that limit virus-driven cell death. Assays were performed under high-confluence (90%), where basal cell proliferation is minimal, thereby reducing the likelihood that changes in cell viability reflect cytostatic effects. To validate the responsiveness and dynamic range of this newly developed assay, we selected four reference compounds with documented anti-coronavirus activity or biological rationale: chloroquine, hydroxychloroquine, molnupiravir, and fluvoxamine.

**Figure 2:**
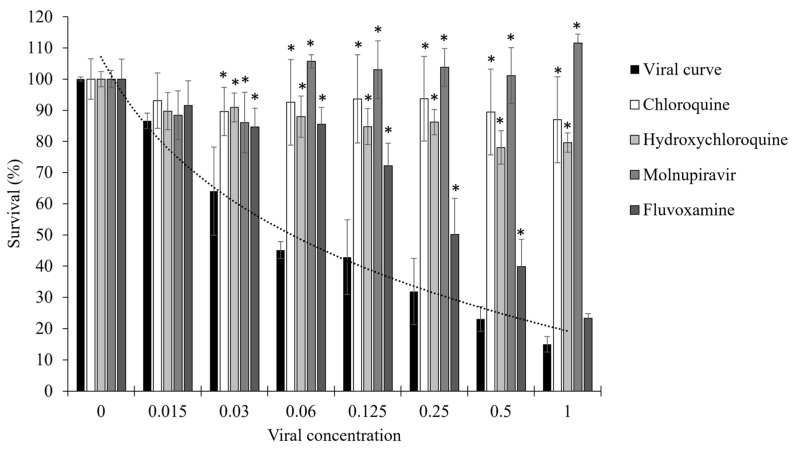
Antiviral activity of positive controls against MRC-5 infected with increasing concentrations of HCoV-OC43. The viral curve corresponds to MRC-5 cells infected with increasing concentrations of HCoV-OC43 without treatment with positive controls. Chloroquine, hydroxychloroquine and molnupiravir provided strong cytoprotection against viral infection, whereas fluvoxamine lost efficacy at higher viral concentrations. For all data, *n* ≥ 3 (biological replicates). * Significantly different from the viral curve (*p* < 0.05). Data are representative of three separate experimental runs.

Chloroquine has been widely reported to exhibit *in vitro* antiviral activity against several viruses, including coronaviruses, and is commonly used as a reference compound in cell-based assays [44,45,46,47,48,49]. Hydroxychloroquine, a hydroxylated analogue, displays similar anti-coronavirus activity profiles [45,47,48]. Molnupiravir (EIDD-2801), a prodrug of N4-hydroxycytidine, inhibits viral replication through lethal mutagenesis during RNA synthesis [50,51,52,53,54,55]. Fluvoxamine, a selective serotonin reuptake inhibitor (SSRI), has been investigated in the context of coronavirus infections and may exert cytoprotective effects through host cell–related mechanisms [56,57,58]. At their respective maximum tolerated concentrations (MTCs), chloroquine (1.25 µg/mL), hydroxychloroquine (1.6 µg/mL), molnupiravir (12.5 µg/mL), and fluvoxamine (12.5 µg/mL) all produced significant (*p* < 0.05) cytoprotective effects compared to the virus-only control (Figure 2). Antiviral protection reached 95% for chloroquine, 78% for hydroxychloroquine, 95% for molnupiravir, and 40% for fluvoxamine (Table 1). These results confirm that the assay reliably detects cytoprotective activity across compounds with distinct mechanisms of action and supports its use for screening complex mixtures such as essential oils and selected constituents.

**Figure 3:**
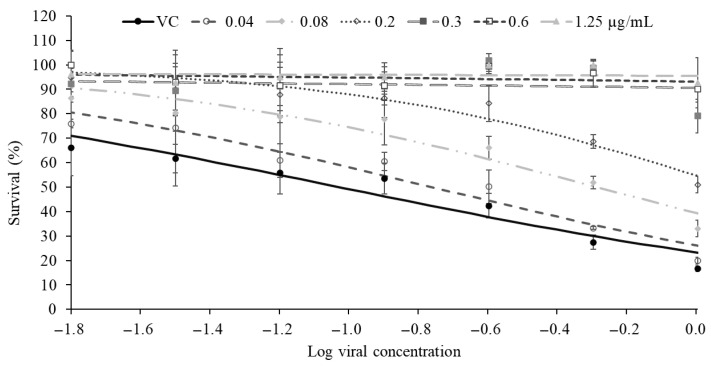
Antiviral activity of chloroquine against MRC-5 cells infected with HCoV-OC43. MRC-5 cells were treated with increasing concentrations of chloroquine (0.04–1.25 µg/mL). The viral curve (VC) corresponds to MRC-5 cells infected with HCoV-OC43 without chloroquine. For all data, *n* ≥ 3 (biological replicates). Data are representative of three separate experimental runs.

**Figure 4:**
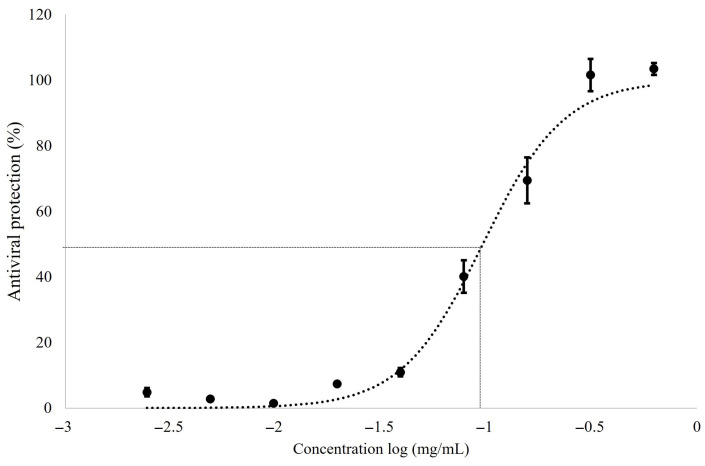
Determination of half maximum effective concentration (EC_50_) of chloroquine against MRC-5 infection with HCoV-OC43. The non-linear sigmoidal regression was obtained using AUC_c_ calculated from chloroquine. Growing concentrations ranged from 0.04 to 1.5 µg/mL. The EC_50_ values (cytoprotection against virus-induced cell death) were derived using a non-linear sigmoidal regression equation. For all data, *n* ≥ 3 (biological replicates). Data are representative of three separate experimental runs.

Reference compounds achieving at least 50% cytoprotection at their maximum tolerated concentration (MTC) were further analyzed to determine EC_50_ values, defined as the concentration producing half-maximal cytoprotection against virus-induced cell death. Fluvoxamine did not reach this threshold and therefore did not allow EC_50_ determination. Dose–response analysis, based on an AUC-derived measure of antiviral protection, enabled robust estimation of EC_50_ values. Chloroquine exhibited the lowest EC_50_ (0.12 ± 0.01 µg/mL), indicating the highest apparent *in vitro* potency, whereas hydroxychloroquine and molnupiravir showed EC_50_ values of 0.5 ± 0. 1 µg/mL (Table 1). Representative data for chloroquine are shown in Figure 3 and Figure 4, and similar trends were observed for the other compounds. Based on its consistent and strong cytoprotective activity, chloroquine was selected as the positive control for subsequent assays. Although chloroquine and hydroxychloroquine exhibited marked *in vitro* activity in this model, their clinical relevance remains limited due to known safety concerns and toxicity risks [59,60].

### 3.3. Antiviral Cytoprotecting of P. balsamifera Bud Essential Oil and Chemical Composition

**Figure 5:**
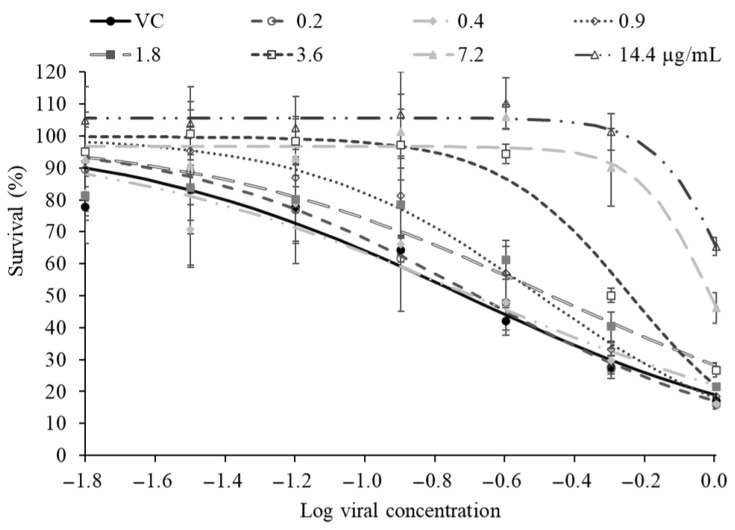
Antiviral activity of *P. balsamifera* essential oil. MRC-5 cells were infected with growing concentrations of HCoV-OC43 and treated at concentrations ranged from between 0.2 and 14.4 µg/mL of *P. balsamifera* essential oil. The EC_50_ value (cytoprotection against virus-induced cell death) was then calculated from the generated dose–response curves. The control is represented by the viral curve (VC). For all data, *n* ≥ 3 (biological replicates). Data are representative of three separate experimental runs.

Van Hoof and Vanden Berghe [61] previously reported antiviral effects of extracts from Populus spp. against viruses such as measles and herpes, supporting the broader potential of poplar-derived products as sources of antiviral agents. Building on this rationale, we evaluated the antiviral cytoprotective activity of *P. balsamifera* bud essential oil using the newly developed MRC-5/HCoV-OC43 assay. As shown in Figure 5, the essential oil provided clear, concentration-dependent protection of MRC-5 cells against HCoV-OC43-induced cytotoxicity. These data demonstrate that *P. balsamifera* bud essential oil effectively protects host cells from HCoV-OC43-driven cell death in this model with a high level of protection at the maximum tolerated concentration (14 µg/mL) of approximately 95% and an EC_50_ of 3.3 ± 0.5 µg/mL (Table 2).

To gain insight into the chemical basis of this antiviral activity, we characterized the essential oil by GC-MS and GC-FID. An annotated GC-MS chromatogram to facilitate visualization of the identified compounds and representative mass spectra of selected compounds are provided in the Supplementary Materials. Twenty-eight constituents present at ≥0.5% were identified, accounting for 83.28% of the total composition (Table 3). The oil was dominated by sesquiterpenes, with α-bisabolol as the major component (22.91%), followed by α-eudesmol (7.67%), δ-cadinene (5.88%), α-amorphene (5.60%), nerolidol (5.52%), and γ-cadinene (3.43%). Consistent with our findings, Isidorov and Vinogorova [62] reported sesquiterpenes (e.g., guaiol, bulnesol, and α-eudesmol) and n-alkanes in *P. balsamifera* buds, and Piochon-Gauthier et al. [63] identified α-bisabolol as a major constituent across multiple *P. balsamifera* bud essential oil samples (18.2–67.7%). Variability in α-bisabolol abundance among studies may reflect differences in geographic origin, climate, soil conditions, genetic background, plant age, and extraction parameters [64,65]. α-Bisabolol has been widely documented for pharmacological properties, including anti-inflammatory and antioxidant activities [63,66,67], and sesquiterpene-rich essential oils have shown antiviral potential in other models [68]. Nevertheless, experimental data on the antiviral contribution of individual major constituents of *P. balsamifera* essential oil remain limited. Nerolidol, for example, has primarily been proposed as a potential anti-SARS-CoV-2 compound *in silico* [69,70], highlighting the need for experimental testing of the principal constituents identified here.

### 3.4. Identification of Active Constituents: α-Bisabolol and Nerolidol

**Figure 6:**
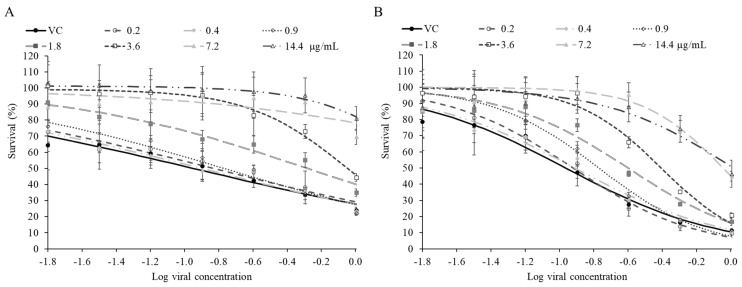
Antiviral activity of α-bisabolol (**A**) and nerolidol (**B**). MRC-5 cells were infected with growing concentrations of HCoV-OC43 and treated at different concentrations of α-bisabolol or nerolidol. Concentrations ranged between 0.2 and 14.4 µg/mL for both molecules. The EC_50_ value (cytoprotection against virus-induced cell death) was then calculated from the generated dose–response curves. The control is represented by the viral curve (VC). For all data, *n* ≥ 3 (biological replicates). Data are representative of three separate experimental runs.

To identify constituents contributing to the antiviral cytoprotective activity of *P. balsamifera* bud essential oil, we selected two abundant sesquiterpene alcohols, α-bisabolol and nerolidol, for individual testing. Both molecules are frequently present at substantial levels in bioactive essential oils and have been associated with diverse pharmacological properties [66,71]. As indicated with the EC_50_ values obtained from the generated concentration–response relationships for both α-bisabolol and nerolidol (Figure 6; Table 2), they produced significant cytoprotective effects against HCoV-OC43-induced cell death. α-Bisabolol displayed strong antiviral cytoprotection with an EC_50_ of 2.7 ± 0.3 µg/mL and approximately 90% protection at the MTC (Figure 6A; Table 2). Although essential oils rich in α-bisabolol (e.g., chamomile essential oil) have previously shown *in vitro* activity against herpes simplex virus (HSV) [66], direct evaluation of the isolated sesquiterpene has, to our knowledge, not been reported. These results therefore provide the first experimental evidence of antiviral cytoprotective activity for α-bisabolol in a human coronavirus model. Nerolidol also showed marked cytoprotective activity with an EC_50_ of 2.6 ± 0.4 µg/mL and approximately 90% protection at the MTC (Figure 6B; Table 2). While nerolidol has been reported to exhibit antiviral effects against mouse polyomavirus [72], the present data appear to constitute the first evidence of antiviral cytoprotection against a human virus in this assay context. Together, these findings support the conclusion that α-bisabolol and nerolidol are key contributors to the antiviral cytoprotective activity of *P. balsamifera* bud essential oil against HCoV-OC43.

Sesquiterpenes and sesquiterpene lactones have been reported to inhibit a broad spectrum of viruses, including influenza A viruses (H1N1, H3N2, H9N2), HBV, HCV, HIV-1, HSV-1, PIV-3, and SARS-CoV-2 [73,74,75]. Reported mechanisms across this chemical class include interference with viral entry, suppression of viral RNA replication, and modulation of viral protein expression [74]. For example, brevilin A inhibits influenza A virus replication without affecting viral entry [75], illustrating that mechanistic diversity is possible even within structurally related terpenes. However, mechanistic and antiviral data for α-bisabolol and nerolidol in the context of coronavirus infection remain scarce. To date, no *in vitro* studies have reported antiviral activity for these compounds when evaluated individually. Available evidence is limited to *in silico* studies, particularly for nerolidol, suggesting potential interactions with SARS-CoV-2 targets. While these computational approaches provide useful hypotheses, they require experimental validation.

In this context, the present study provides the first experimental evidence of antiviral cytoprotective activity for these compounds in a human coronavirus model, thereby contributing compound-specific data to a field that has so far relied primarily on class-level observations. Because our readout is based on cytoprotection, future studies should incorporate orthogonal virological endpoints such as time-of-addition experiments, viral RNA/protein quantification, and infectious titer measurements to determine whether these sesquiterpenes act primarily on entry, replication, and/or host protective pathways.

## 4. Conclusions

The continued emergence and re-emergence of viral diseases underscore the need to expand the antiviral discovery pipeline and to identify new bioactive chemical scaffolds. In this work, we developed a sensitive, BSL-2-compatible cytoprotection assay that enables rapid screening of complex extracts and purified compounds against HCoV-OC43. Using this platform, we demonstrate for the first time that *P. balsamifera* bud essential oil exhibits antiviral cytoprotective activity against a human coronavirus. Chemical profiling by GC-MS/GC-FID further revealed a sesquiterpene-rich composition, and two abundant constituents, α-bisabolol and nerolidol, were identified as active compounds in this model. Interestingly, although α-bisabolol and nerolidol exhibited lower EC_50_ values, the essential oil showed stronger overall cytoprotective effects at the MTC, suggesting that additional constituents and/or synergistic interactions may contribute to the observed activity. To our knowledge, these results provide the first experimental evidence of antiviral cytoprotection by α-bisabolol and nerolidol against a human coronavirus in a cell-based assay context.

It should be noted that these results are based on a cytoprotection assay measuring virus-induced cytopathic effects on cellular viability and performed under high-confluence conditions designed to minimize active cell proliferation. Thus, the developed assay does not allow direct quantification of viral replication or mechanistic discrimination between antiviral activity and host cell-mediated responses. Despite this limitation, the results highlight *P. balsamifera* as a promising source of antiviral bioactive compounds. Compared to other *Populus* species, although direct comparative antiviral data remain scarce, the relatively high abundance of bioactive sesquiterpenes such as α-bisabolol and nerolidol, combined with the strong cytoprotective activity observed, suggests that *P. balsamifera* may represent a particularly promising source of antiviral compounds within the genus. These findings open new perspectives for additional investigation of bud-derived essential oils as underexplored antiviral natural products. Future studies should focus on elucidating the mechanisms of action, including potential effects on viral entry and post-entry events. Finally, comparative studies with other *Populus* species and a wider range of natural products would further contextualize the bioactivity of *P. balsamifera* within plant-derived antiviral research.

## Figures and Tables

**Table 1:** Antiviral activity of positive controls against MRC-5 infection induced by HCoV-OC43.

	Chloroquine	Hydroxychloroquine	Molnupiravir	Fluvoxamine
AUC_v_ ^a^	196 ± 23	221 ± 45	246 ± 64	219 ± 34
MTC (µg/mL) ^b^	1.25	1.6	12.5	12.5
AUC_c_ ^c^	485 ± 31 *	440 ± 25 *	487 ± 33 *	332 ± 31 *
Protection ^d^ (%)	95	78	95	40
EC_50_ ^e^ (µg/mL)	0.11 ± 0.01	0.5 ± 0.1	0.5 ± 0.1	ND

Data are representative of three separate experimental runs. For all data, *n* ≥ 3 (biological replicates). * Significantly different from the respective viral curve for each treatment (*p* < 0.05). ^a^ AUC_v_: area under the cell-viability curve for HCoV-OC43 infection in the absence of treatment (virus alone). ^b^ MTC (maximum tolerated concentration): the highest concentration tested that maintains ≥80% cell viability. ^c^ AUC_c_: area under the cell-viability curve for HCoV-OC43 infection in the presence of the tested compound (virus + compound). ^d^ Antiviral protection (%) at the maximum tolerated concentration (MTC). ^e^ Concentration producing a half-maximal cytoprotective effect against virus-induced cell death. ND = not determined.

**Table 2:** Antiviral activity of essential oil from *P. balsamifera* buds and constituents against MRC-5 infection induced by HCoV-OC43.

	Essential Oil	α-Bisabolol	Nerolidol	Chloroquine ^a^
AUC_v_ ^b^	229 ± 24	211 ± 24	237 ± 28	192 ± 51
MTC (µg/mL) ^c^	14	14	55	0.625
AUC_c_ ^d^	486 ± 34 *	471 ± 41 *	474 ± 28 *	510 ± 9 *
Protection ^e^ (%)	95	90	90	100
EC_50_ ^f^ (µg/mL)	3.3 ± 0.5	2.7 ± 0.3	2.6 ± 0.4	0.10 ± 0.01

* Significantly different from the respective viral curve for each treatment (*p* < 0.05). Data are representative of three separate experimental runs. For all data, *n* ≥ 3 (biological replicates). ^a^ Positive control ^b^ AUC_v_: area under the cell-viability curve for HCoV-OC43 infection in the absence of treatment (virus alone). ^c^ MTC (maximum tolerated concentration): the highest concentration tested that maintains ≥ 80% cell viability. ^d^ AUC_c_: area under the cell-viability curve for HCoV-OC43 infection in the presence of the tested compound (virus + compound). ^e^ Antiviral protection (%) at the maximum tolerated concentration (MTC). ^f^ Concentration producing a half-maximal cytoprotective effect against virus-induced cell death.

**Table 3:** Chemical composition of the essential oil from *P. balsamifera* buds.

Peak No.	Identified Components			Relative Concentration (%)	*m*/*z* Values (Ordered by Abundance)
	RI	Name	Identification		
1	1023	1,8-Cineole	a,b	1.48	43, 81, 108, 71, 84, 111, 69, 154, 93, 139
2	1392	Ylangene	a,b	0.77	105, 119, 93, 120, 161, 91, 92, 107, 41, 121
3	1433	*Trans*-α-Bergamotene	a,b	2.15	93, 119, 91, 41, 69, 107, 79, 77, 105, 55
4	1440	*Trans*-β-Farnesene	a,b	1.71	69, 93, 41, 133, 79, 67, 91, 81, 120, 161
5	1475	4-epi-α-acoradiene	a,b	0.87	119, 93, 121, 105, 79, 91, 77, 41, 107, 67
6	1474	γ-Amorphene	a,b	1.06	161, 105, 119, 91, 93, 79, 133, 77, 41, 204
7	1479	α-Amorphene	a,b	5.60	105, 119, 93, 91, 121, 161, 94, 204, 79, 77
8	1472	Curcumene	a,b	2.21	119, 132, 105, 91, 131, 145, 41, 120, 202, 117
9	1491	β-Cadinene	a	0.67	173, 158, 189, 161, 143, 204, 105, 91, 128, 133
10	1494	γ-Muurolene	a,b	1.37	161, 105, 119, 93, 91, 79, 133, 41, 120, 81
11	1490	α-Muurolene	a	1.82	161, 105, 119, 91, 204, 81, 93, 133, 41, 79
12	1500	β-Bisabolene	a,b	0.83	69, 93, 41, 79, 67, 91, 94, 109, 107, 119
13	1505	δ-Cadinene	a	5.88	161, 119, 105, 91, 93, 79, 41, 77, 204, 133
14	1505	γ-Cadinene	a,b	3.43	161, 119, 204, 134, 105, 91, 81, 162, 189, 133
15	1535	Selina-3,7(11)-diene	a,b	2.93	161, 122, 107, 204, 105, 91, 93, 81, 119, 41
16	1548	α-Copaen-11-ol	a	0.64	59, 150, 119, 91, 94, 159, 93, 106, 147, 134
17	1539	α-Calacorene	a,b	1.10	157, 142, 141, 156, 200, 158, 115, 143, 128, 155
18	1506	*Cis*-α-Bisabolene	a,b	2.27	93, 119, 121, 91, 80, 79, 109, 41, 107, 92
19	1545	Nerolidol	a,b	5.52	69, 93, 41, 107, 71, 43, 81, 55, 67, 136
20	1595	α-Cedrol	a	2.97	151, 69, 67, 93, 55, 95, 41, 68, 81, 109
21	1596	Fokienol	a	0.50	93, 119, 71, 107, 91, 43, 105, 79, 55, 81
22	1628	Agarospirol	a,b	0.72	161, 59, 119, 105, 91, 93, 107, 204, 135, 79
23	1627	γ-Eudesmol	a,b	3.07	161, 189, 204, 105, 91, 133, 59, 107, 93, 81
24	1628	*Trans*-Cadinol	a,b	1.01	161, 204, 43, 105, 81, 95, 119, 79, 162, 121
25	1644	β-Eudesmol	a,b	1.27	59, 149, 164, 109, 108, 79, 93, 95, 81, 91
26	1643	α-Eudesmol	a	7.67	59, 149, 161, 204, 189, 93, 91, 95, 109, 107
27	1653	β-Bisabolol	a,b	0.84	82, 93, 41, 69, 111, 121, 67, 55, 83, 119
28	1683	α-Bisabolol	a,b	22.91	109, 119, 69, 43, 93, 41, 95, 67, 121, 71
			Total identified (%)	83.28	

(a) Retention indices (RIs) on the non-polar DB-5 column were calculated from gas chromatography–mass spectrometry (GC–MS) data using the retention times of n-alkanes (C7–C40). These values were compared with literature data and our in-house database. (b) Compound identification was done by comparing mass spectra with reference library data, applying a reverse match factor ≥ 900. Representative mass spectra are provided in the Supplementary Materials.

## Data Availability

Data is contained within the article.

## Supplementary Materials

The following supporting information can be downloaded at: https://www.mdpi.com/article/10.3390/molecules31091496/s1.

## Abbreviations

The following abbreviations are used in this manuscript:

HSV	Herpes Simplex Virus
HIV	Human Immunodeficiency Virus
MEM	Minimal Essential Medium Eagle
FBS	Fetal Bovine Serum
ATCC	American Type Culture Collection
HBSS	Hank’s Balanced Salt Solution
PBS	Phosphate-Buffered Saline
MTC	Maximum Tolerated Concentration
AUC	Area Under the Curve
EC_50_	Half-Maximal Effective Concentration
GC-MS	Gas Chromatography-Mass Spectrometry
GC-FID	Gas Chromatrography with Flame Ionization Detection
BSL-2	Biosafety Level 2
NHC	N4-Hydroxycytidine
SSRI	Selective Serotonin Reuptake Inhibitor
HBV	Hepatitis B Virus
HCV	Hepatitis C Virus
PIV-3	Parainfluenza Virus type 3

## References

[1] Kesheh M.M., Hosseini P., Soltani S., Zandi M. (2022). An overview on the seven pathogenic human coronaviruses. Rev. Med. Virol..

[2] Liu D.X., Liang J.Q., Fung T.S. (2021). Human coronavirus-229E, -OC43, -NL63, and -HKU1 (*Coronaviridae*). Encycl. Virol..

[3] Romanowski V. (2013). Current Issues in Molecular Virology: Viral Genetics and Biotechnological Applications.

[4] Alves M.C.S., da Silva R.C.C., de Leitão-Júnior S.S.P., de Balbino V.Q. (2025). Therapeutic approaches for COVID-19: A review of antiviral treatments, immunotherapies, and emerging interventions. Adv. Ther..

[5] Bryan-Marrugo O.L., Ramos-Jiménez J., Barrera-Saldaña H., Rojas-Martínez A., Vidaltamayo R., Rivas-Estilla A.M. (2015). History and progress of antiviral drugs: From acyclovir to direct-acting antiviral agents (DAAs) for Hepatitis C. Med. Univ..

[6] Pardi N., Weissman D. (2020). Development of vaccines and antivirals for combating viral pandemics. Nat. Biomed. Eng..

[7] Landry M.L., Stanat S., Biron K., Brambilla D., Britt W., Jokela J., Chou S., Drew W.L., Erice A., Gilliam B. (2000). A standardized plaque reduction assay for determination of drug susceptibilities of cytomegalovirus clinical isolates. Antimicrob. Agents Chemother..

[8] Prichard M.N., Turk S.R., Coleman L.A., Engelhardt S.L., Shipman C., Drach J.C. (1990). A microtiter virus yield reduction assay for the evaluation of antiviral compounds against human cytomegalovirus and herpes simplex virus. J. Virol. Methods.

[9] Zhang Q., Liu Z., Mi Z., Li X., Jia P., Zhou J., Yin X., You X., Yu L., Guo F. (2011). High-throughput assay to identify inhibitors of Vpu-mediated down-regulation of cell surface BST-2. Antivir. Res..

[10] Green N., Ott R.D., Isaacs R.J., Fang H. (2008). Cell-based assays to identify inhibitors of viral disease. Expert Opin. Drug Discov..

[11] Savoie C., Lippé R. (2022). Optimizing human coronavirus OC43 growth and titration. PeerJ.

[12] Schmidtke M., Schnittler U., Jahn B., Dahse H.M., Stelzner A. (2001). A rapid assay for evaluation of antiviral activity against coxsackie virus B3, influenza virus A, and herpes simplex virus type 1. J. Virol. Methods.

[13] Praditya D.F., Waluyo D., Nozaki T. (2025). Reporter-expressing viruses for antiviral drug discovery research. Front. Cell. Infect. Microbiol..

[14] Wang J., Zhu Q., Xing X., Sun D. (2024). A mini-review on the common antiviral drug targets of coronavirus. Microorganisms.

[15] Bowden-Reid E., Moles E., Kelleher A., Ahlenstiel C. (2025). Harnessing antiviral RNAi therapeutics for pandemic viruses: SARS-CoV-2 and HIV. Drug Deliv. Transl. Res..

[16] Masyita A., Mustika Sari R., Dwi Astuti A., Yasir B., Rahma Rumata N., Emran T.B., Nainu F., Simal-Gandara J. (2022). Terpenes and terpenoids as main bioactive compounds of essential oils, their roles in human health and potential application as natural food preservatives. Food Chem. X.

[17] Önder S., Periz Ç.D., Ulusoy S., Erbaş S., Önder D., Tonguç M. (2024). Chemical composition and biological activities of essential oils of seven Cultivated *Apiaceae* species. Sci. Rep..

[18] Pezantes-Orellana C., German Bermúdez F., Matías De la Cruz C., Montalvo J.L., Orellana-Manzano A. (2024). Essential oils: A systematic review on revolutionizing health, nutrition, and omics for optimal well-being. Front. Med..

[19] Ćavar Zeljković S., Schadich E., Džubák P., Hajdúch M., Tarkowski P. (2022). Antiviral activity of selected lamiaceae essential oils and their monoterpenes against SARS-CoV-2. Front. Pharmacol..

[20] Li D.-Y., Donadu M.G., Shue T., Dangas G., Athanasiadis A., Lan S., Wen X., Battah B., Zanetti S., Mazzarello V. (2024). *Myrtus communis* L. Essential oil exhibits antiviral activity against coronaviruses. Pharmaceuticals.

[21] Wani A.R., Yadav K., Khursheed A., Rather M.A. (2021). An updated and comprehensive review of the antiviral potential of essential oils and their chemical constituents with special focus on their mechanism of action against various influenza and coronaviruses. Microb. Pathog..

[22] Iqhrammullah M., Rizki D.R., Purnama A., Duta T.F., Harapan H., Idroes R., Ginting B. (2023). Antiviral molecular targets of essential oils against SARS-CoV-2: A systematic review. Sci. Pharm..

[23] Licata A., Seidita A., Como S., de Carlo G., Cammilleri M., Bonica R., Soresi M., Veronese N., Chianetta R., Citarrella R. (2025). Herbal and dietary supplements as adjunctive treatment for mild SARS-CoV-2 infection in Italy. Nutrients.

[24] Poaty B., Lahlah J., Porqueres F., Bouafif H. (2015). Composition, antimicrobial and antioxidant activities of seven essential oils from the North American boreal forest. World J. Microbiol. Biotechnol..

[25] Marie-Victorin F., Rouleau E., Brouillet L. (1995). Flore Laurentienne.

[26] Bélanger A., Grenier A., Simard F., Gendreau I., Pichette A., Legault J., Pouliot R. (2020). Dihydrochalcone derivatives from *Populus balsamifera* L. Buds for the treatment of psoriasis. Int. J. Mol. Sci..

[27] Guleria I., Kumari A., Lacaille-Dubois M.-A., Nishant Kumar V., Saini A.K., Dhatwalia J., Lal S. (2022). A review on the genus *Populus*: A potential source of biologically active compounds. Phytochem. Rev..

[28] Holloway P.S., Alexander G. (1990). Ethnobotany of the Fort Yukon Region, Alaska. Econ. Bot..

[29] Simard F., Gauthier C., Chiasson É., Lavoie S., Mshvildadze V., Legault J., Pichette A. (2015). Antibacterial balsacones J–M, hydroxycinnamoylated dihydrochalcones from *Populus balsamifera* buds. J. Nat. Prod..

[30] Moerman D.E. (2009). Native American Medicinal Plants: An Ethnobotanical Dictionary.

[31] Uprety Y., Asselin H., Dhakal A., Julien N. (2012). Traditional use of medicinal plants in the boreal forest of Canada: Review and perspectives. J. Ethnobiol. Ethnomed..

[32] Harbilas D., Brault A., Vallerand D., Martineau L.C., Saleem A., Arnason J.T., Musallam L., Haddad P.S. (2012). *Populus balsamifera* L. (Salicaceae) mitigates the development of obesity and improves insulin sensitivity in a diet-induced obese mouse model. J. Ethnopharmacol..

[33] Trabelsi K., Majoul S., Rourou S., Kallel H. (2012). Development of a measles vaccine production process in MRC-5 cells grown on Cytodex1 microcarriers and in a stirred bioreactor. Appl. Microbiol. Biotechnol..

[34] Hafidh R.R., Abdulamir A.S., Abu Bakar F., Sekawi Z., Jahansheri F., Jalilian F.A. (2015). Novel antiviral activity of mung bean sprouts against respiratory syncytial virus and herpes simplex virus −1: An in vitro study on virally infected Vero and MRC-5 cell lines. BMC Complement. Altern. Med..

[35] O’Brien J., Wilson I., Orton T., Pognan F. (2000). Investigation of the alamar blue (resazurin) fluorescent dye for the assessment of mammalian cell cytotoxicity. Eur. J. Biochem..

[36] Adams R.P. (2007). Identification of Essential Oil Components by Gas Chromatography/Mass Spectrometry.

[37] (NIST) NIoSaT (2017). NIST Mass Spectral Database.

[38] Hochmuth D.H. (2004). MassFinder 4.

[39] Lamers M.M., Haagmans B.L. (2022). SARS-CoV-2 pathogenesis. Nat. Rev. Microbiol..

[40] Bresson S., Sani E., Armatowska A., Dixon C., Tollervey D. (2025). The transcriptional and translational landscape of HCoV-OC43 infection. PLoS Pathog..

[41] Min J.S., Kim D.E., Jin Y.-H., Kwon S. (2020). Kurarinone inhibits HCoV-OC43 infection by impairing the virus-induced autophagic flux in MRC-5 human lung cells. J. Clin. Med..

[42] Kim D.E., Min J.S., Jang M.S., Lee J.Y., Shin Y.S., Song J.H., Kim H.R., Kim S., Jin Y.H., Kwon S. (2019). Natural bis-benzylisoquinoline alkaloids-tetrandrine, fangchinoline, and cepharanthine, inhibit human coronavirus oc43 infection of mrc-5 human lung cells. Biomolecules.

[43] Chatow L., Nudel A., Eyal N., Lupo T., Ramirez S., Zelinger E., Nesher I., Boxer R. (2024). Terpenes and cannabidiol against human corona and influenza viruses–Anti-inflammatory and antiviral in vitro evaluation. Biotechnol. Rep..

[44] Ribaudo G., Coghi P., Yang L.J., Ng J.P.L., Mastinu A., Memo M., Wong V.K.W., Gianoncelli A. (2022). Computational and experimental insights on the interaction of artemisinin, dihydroartemisinin and chloroquine with SARS-CoV-2 spike protein receptor-binding domain (RBD). Nat. Prod. Res..

[45] Malik P., Jain S., Jain P., Kumawat J., Dwivedi J., Kishore D. (2022). A comprehensive update on the structure and synthesis of potential drug targets for combating the coronavirus pandemic caused by SARS-CoV-2. Arch. Pharm..

[46] Tabatabaei S.N., Keykhaee Z., Nooraei S., Ayati M.A., Behzadmand M., Azimi S., Eskati F., Ahmadian G. (2025). SARS-CoV-2 and coronaviruses: Understanding transmission, impact, and strategies for prevention and treatment. Drugs Drug Candidates.

[47] Yuan Z., Pavel M.A., Wang H., Kwachukwu J.C., Mediouni S., Jablonski J.A., Nettles K.W., Reddy C.B., Valente S.T., Hansen S.B. (2022). Hydroxychloroquine blocks SARS-CoV-2 entry into the endocytic pathway in mammalian cell culture. Commun. Biol..

[48] Ni Y., Liao J., Qian Z., Wu C., Zhang X., Zhang J., Xie Y., Jiang S. (2022). Synthesis and evaluation of enantiomers of hydroxychloroquine against SARS-CoV-2 in vitro. Bioorg. Med. Chem..

[49] Velásquez P.A., Hernandez J.C., Galeano E., Hincapié-García J., Rugeles M.T., Zapata-Builes W. (2024). Effectiveness of drug repurposing and natural products against SARS-CoV-2: A comprehensive review. Clin. Pharmacol. Adv. Appl..

[50] Li P., Wang Y., Lavrijsen M., Lamers M.M., de Vries A.C., Rottier R.J., Bruno M.J., Peppelenbosch M.P., Haagmans B.L., Pan Q. (2022). SARS-CoV-2 Omicron variant is highly sensitive to molnupiravir, nirmatrelvir, and the combination. Cell Res..

[51] Yip A.J.W., Low Z.Y., Chow V.T.K., Lal S.K. (2022). Repurposing molnupiravir for COVID-19: The mechanisms of antiviral activity. Viruses.

[52] Pandey K., Acharya A., Pal D., Jain P., Singh K., Durden D.L., Kutateladze T.G., Deshpande A.J., Byrareddy S.N. (2024). SRX3177, a CDK4/6-PI3K-BET inhibitor, in combination with an RdRp inhibitor, Molnupiravir, or an entry inhibitor MU-UNMC-2, has potent antiviral activity against the Omicron variant of SARS-CoV-2. Antivir. Res..

[53] Li P., Wang Y., Lamers M.M., Lavrijsen M., Iriondo C., de Vries A.C., Rottier R.J., Peppelenbosch M.P., Haagmans B.L., Pan Q. (2022). Recapitulating infection, thermal sensitivity and antiviral treatment of seasonal coronaviruses in human airway organoids. eBioMedicine.

[54] Negru P.A., Radu A.-F., Vesa C.M., Behl T., Abdel-Daim M.M., Nechifor A.C., Endres L., Stoicescu M., Pasca B., Tit D.M. (2022). Therapeutic dilemmas in addressing SARS-CoV-2 infection: Favipiravir versus Remdesivir. Biomed. Pharmacother..

[55] Brown A.J., Won J.J., Graham R.L., Dinnon K.H., Sims A.C., Feng J.Y., Cihlar T., Denison M.R., Baric R.S., Sheahan T.P. (2019). Broad spectrum antiviral remdesivir inhibits human endemic and zoonotic deltacoronaviruses with a highly divergent RNA dependent RNA polymerase. Antivir. Res..

[56] Hashimoto Y., Suzuki T., Hashimoto K. (2022). Mechanisms of action of fluvoxamine for COVID-19: A historical review. Mol. Psychiatry.

[57] Zhou Q., Zhao G., Pan Y., Zhang Y., Ni Y. (2024). The efficacy and safety of fluvoxamine in patients with COVID-19: A systematic review and meta-analysis from randomized controlled trials. PLoS ONE.

[58] Fred S.M., Kuivanen S., Ugurlu H., Casarotto P.C., Levanov L., Saksela K., Vapalahti O., Castrén E. (2022). Antidepressant and antipsychotic drugs reduce viral infection by SARS-CoV-2 and fluoxetine shows antiviral activity against the novel variants in vitro. Front. Pharmacol..

[59] Della Porta A., Bornstein K., Coye A., Montrief T., Long B., Parris M.A. (2020). Acute chloroquine and hydroxychloroquine toxicity: A review for emergency clinicians. Am. J. Med..

[60] Rao R., Singh J. (2025). Innate immune and endoplasmic reticulum unfolded protein response pathways protect Caenorhabditis elegans against chloroquine toxicity. J. Biosci..

[61] Van Hoof L., Vanden Berghe D.A., Vlietinck A.J. (1980). Screening of poplar trees for antibacterial, antifungal and antiviral activity. Biol. Plant..

[62] Isidorov V.A., Vinogorova V.T. (2003). GC-MS analysis of compounds extracted from buds of *Populus balsamifera* and *Populus nigra*. Z. Naturforschung C.

[63] Piochon-Gauthier M., Legault J., Sylvestre M., Pichette A. (2014). The essential oil of *Populus balsamifera* buds: Its chemical composition and cytotoxic activity. Nat. Prod. Commun..

[64] Abdelmohsen U.R., Elmaidomy A.H. (2025). Exploring the therapeutic potential of essential oils: A review of composition and influencing factors. Front. Nat. Prod..

[65] Jaradat N., Barkat A., Khasati A., Abualhasan M. (2025). Variations of the chemical components and biological activities of *Thymus capitatus* essential oil from three regions in Palestine. Sci. Rep..

[66] Kamatou G.P.P., Viljoen A.M. (2009). A review of the application and pharmacological properties of α-bisabolol and α-bisabolol-rich oils. J. Am. Oil Chem. Soc..

[67] Beegam S., Zaaba N.E., Elzaki O., Nemmar A. (2025). α-Bisabolol alleviates diesel exhaust particle-induced lung injury and mitochondrial dysfunction by regulating inflammatory, oxidative stress, and apoptotic biomarkers through the c-Jun N-terminal kinase signaling pathway. Front. Pharmacol..

[68] Dunkić V., Vuko E., Bezić N., Kremer D., Ruščić M. (2013). Composition and antiviral activity of the essential oils of *Eryngium alpinum* and *E. amethystinum*. Chem. Biodivers..

[69] Costa R.B., Martins R.M., Lima G.S.d., Stamford T.C., Tadei W.P., Maciel M.A.M., do Rêgo A.C.M., Xavier-Júnior F.H. (2022). Molecular docking in silico analysis of Brazilian essential oils against host targets and SARS-CoV-2 proteins. J. Braz. Chem. Soc..

[70] Habibzadeh S., Zohalinezhad M.E. (2022). Evaluation of the inhibitory activities of *Ferula gummosa* bioactive compounds against the druggable targets of SARS-CoV-2: Molecular docking simulation. Biointerface Res. Appl. Chem..

[71] Chan W.-K., Tan L.T., Chan K.-G., Lee L.-H., Goh B.-H. (2016). Nerolidol: A sesquiterpene alcohol with multi-faceted pharmacological and biological activities. Molecules.

[72] Ryabchenko B., Tulupova E., Schmidt E., Wlcek K., Buchbauer G., Jirovetz L. (2008). Investigation of anticancer and antiviral properties of selected aroma samples. Nat. Prod. Commun..

[73] Zhang X., He J., Huang W., Huang H., Zhang Z., Wang J., Yang L., Wang G., Wang Y., Li Y.-L. (2018). Antiviral activity of the sesquiterpene lactones from *Centipeda minima* against influenza A virus in vitro. Nat. Prod. Commun..

[74] Amen Y., Abdelwahab G., Heraiz A.A., Sallam M., Othman A. (2025). Exploring sesquiterpene lactones: Structural diversity and antiviral therapeutic insights. RSC Adv..

[75] Zhang X., Xia Y., Yang L., He J., Li Y., Xia C. (2019). Brevilin A, a sesquiterpene lactone, inhibits the replication of influenza A virus in vitro and in vivo. Viruses.

