# Supplementary material for: Antiviral Activity of Essential Oil from Populus balsamifera L. Buds and Its Major Compounds Against Betacoronavirus HCoV-OC43 Using a Sensitive Cytoprotection Assay
Source: Molecules. 2026 Apr 30;31(9):1496. doi: 10.3390/molecules31091496 (PMC13165263; doi:10.3390/molecules31091496)

Spectrum Plot Report

Agilent | Trusted Answers

Name	PopB	Rack Pos.		Instrument	GCMSD	Operator	Heloise
Inj. Vol. (ul)	0	Plate Pos.		IRM Status			
Data File	POPB.D	Method (Acq)	HE-HC.M	Comment		Acq. Time (Local)	2023-05-23 5:44:31 PM (UTC-04:00)

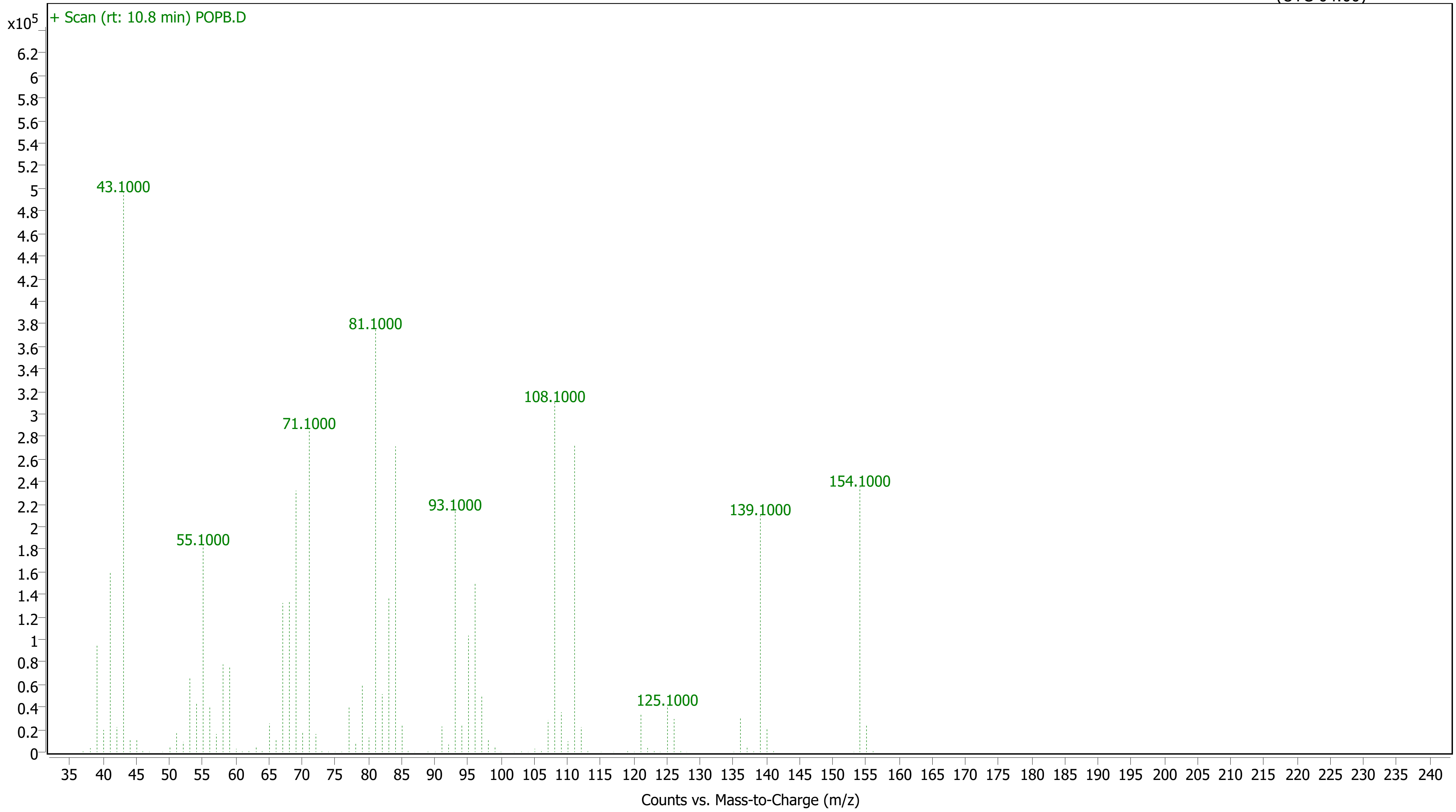

Spectrum Plot Report

Name	PopB	Rack Pos.		Instrument	GCMSD	Operator	Heloise
Inj. Vol. (ul)	0	Plate Pos.		IRM Status			
Data File	POPB.D	Method (Acq)	HE-HC.M	Comment		Acq. Time (Local)	2023-05-23 5:44:31 PM (UTC-04:00)

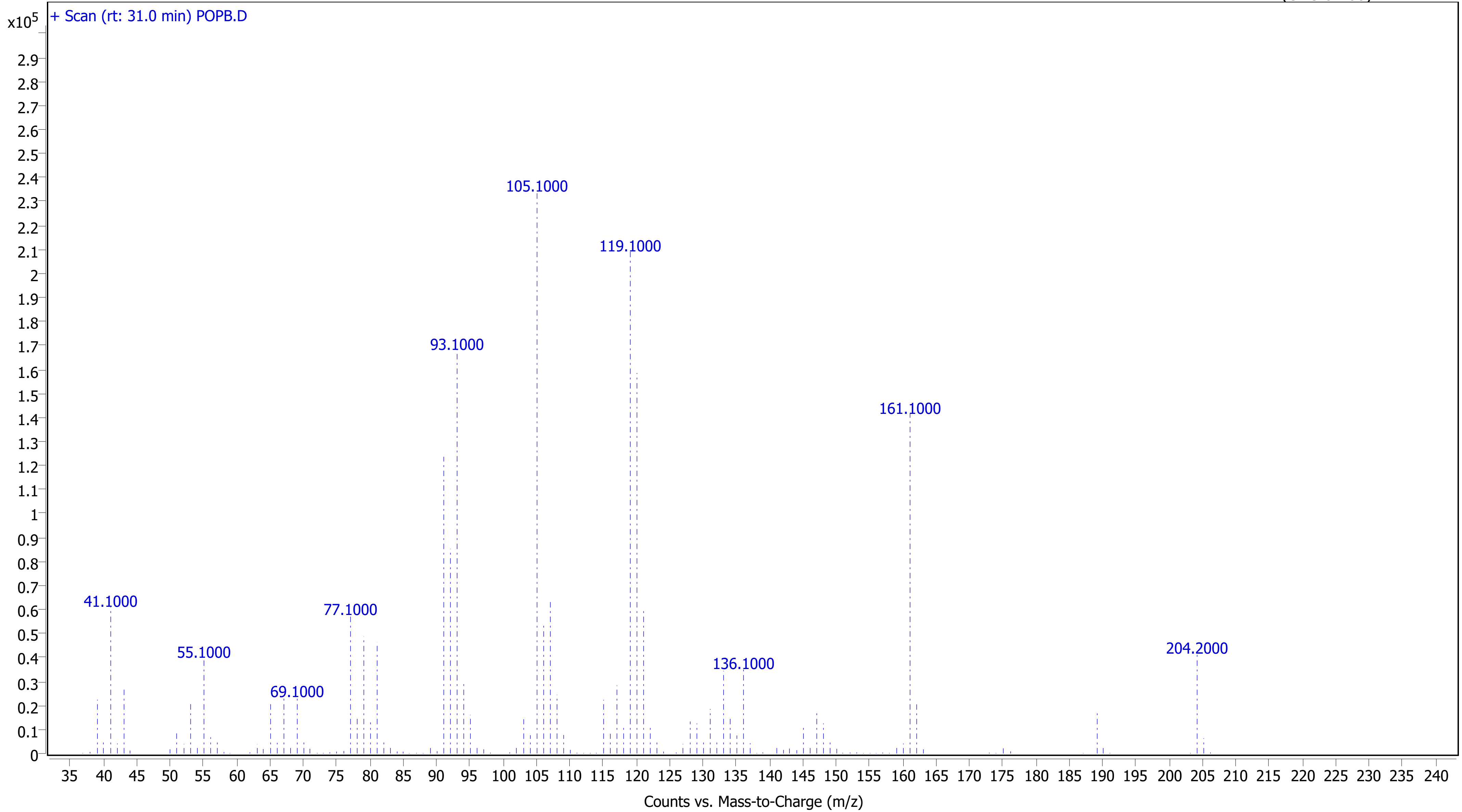

Generated at 6:02 PM on 2026-04-17

Spectrum Plot Report

Name	PopB	Rack Pos.		Instrument	GCMSD	Operator	Heloise
Inj. Vol. (ul)	0	Plate Pos.		IRM Status			
Data File	POPB.D	Method (Acq)	HE-HC.M	Comment		Acq. Time (Local)	2023-05-23 5:44:31 PM (UTC-04:00)

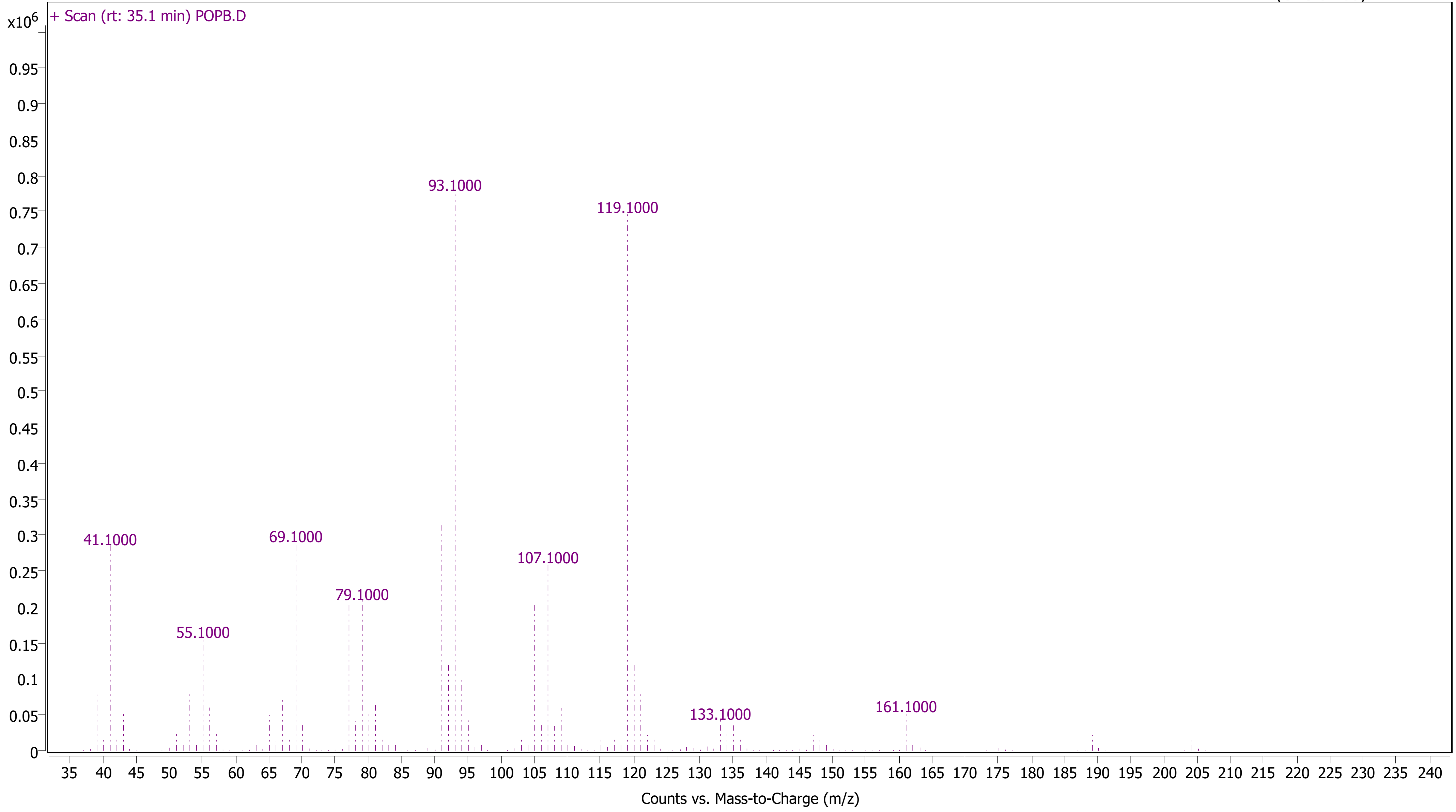

Spectrum Plot Report

Name	PopB	Rack Pos.		Instrument	GCMSD	Operator	Heloise
Inj. Vol. (ul)	0	Plate Pos.		IRM Status			
Data File	POPB.D	Method (Acq)	HE-HC.M	Comment		Acq. Time (Local)	2023-05-23 5:44:31 PM (UTC-04:00)

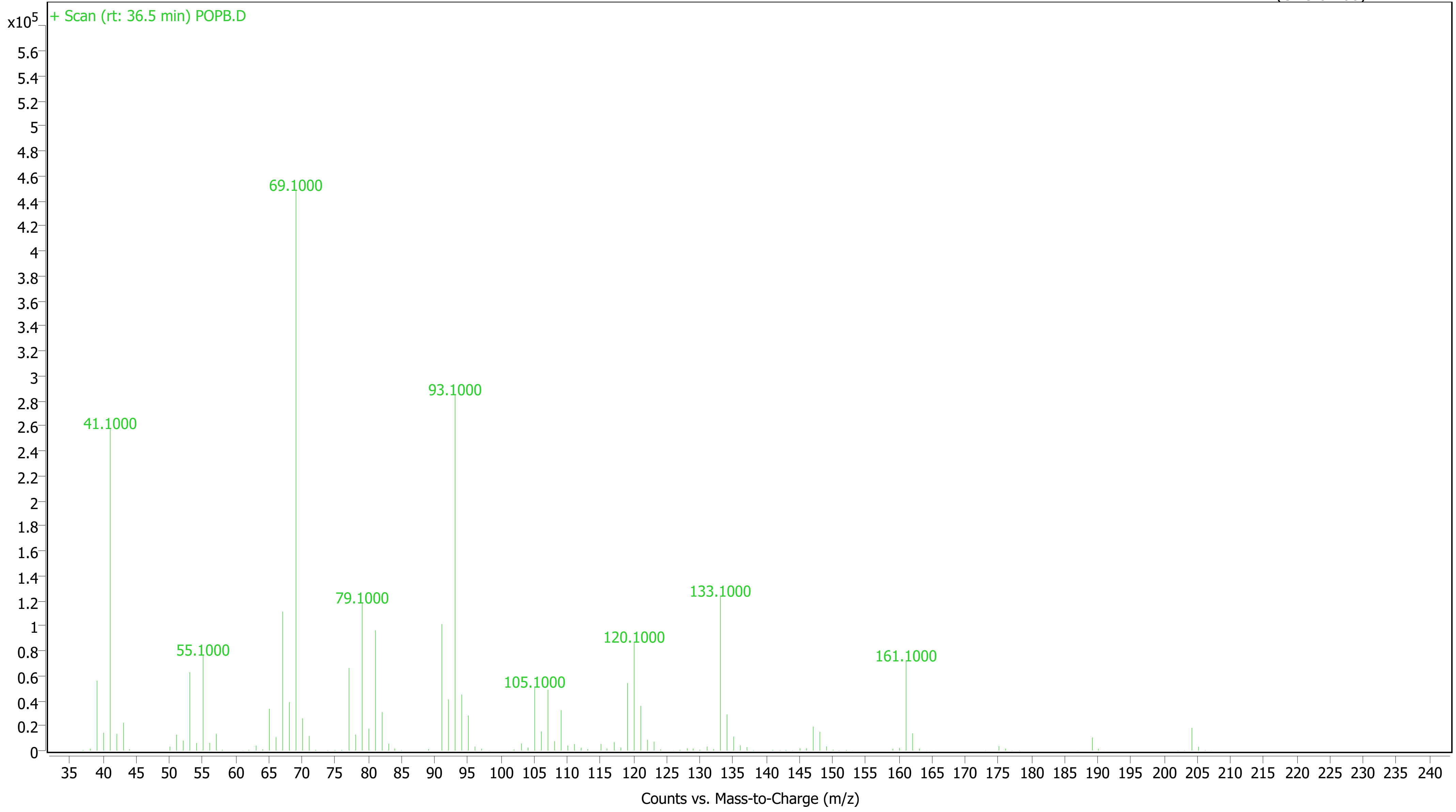

Spectrum Plot Report

Name	PopB	Rack Pos.		Instrument	GCMSD	Operator	Heloise
Inj. Vol. (ul)	0	Plate Pos.		IRM Status			
Data File	POPB.D	Method (Acq)	HE-HC.M	Comment		Acq. Time (Local)	2023-05-23 5:44:31 PM (UTC-04:00)

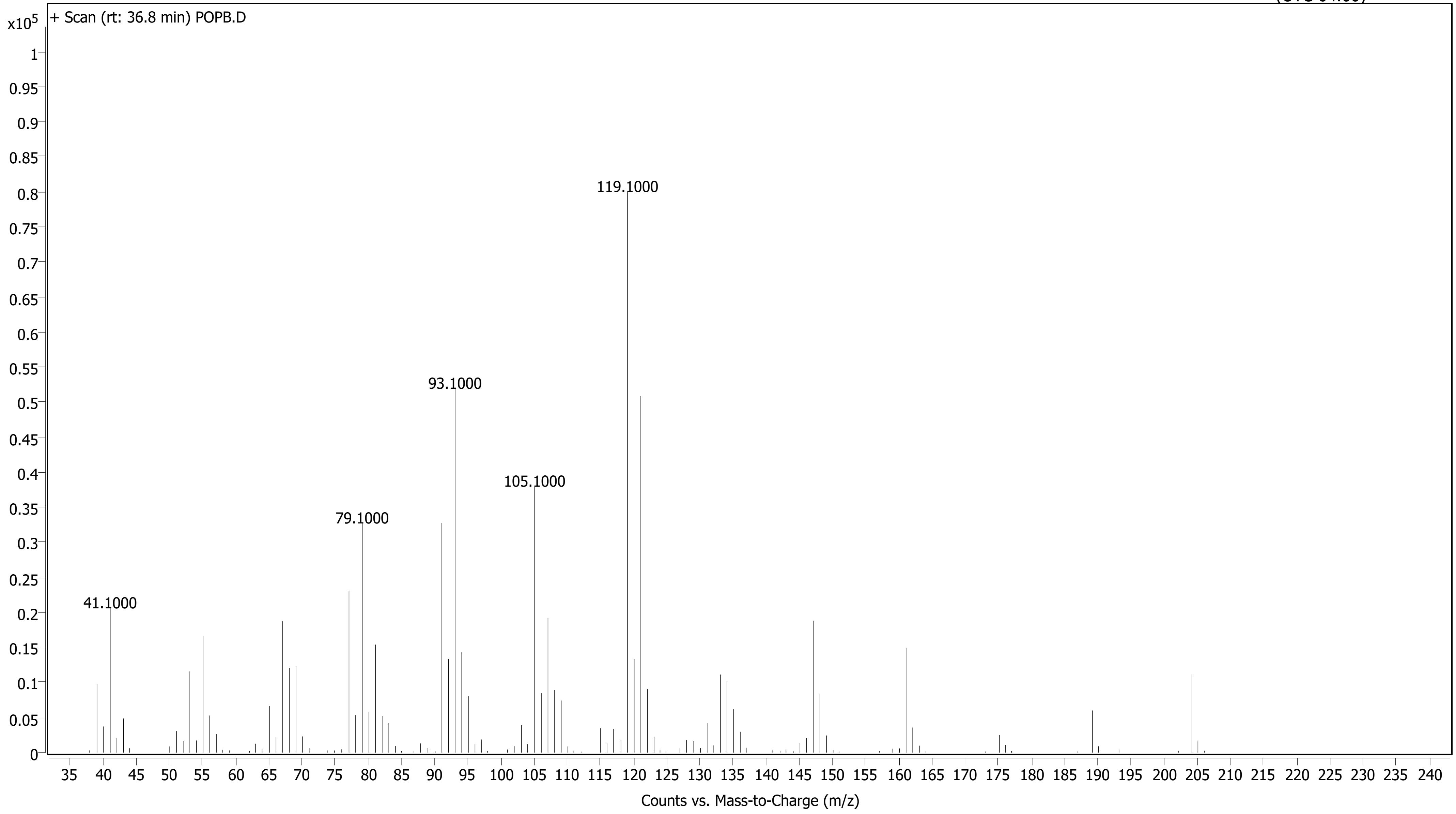

Spectrum Plot Report

Name	PopB	Rack Pos.		Instrument	GCMSD	Operator	Heloise
Inj. Vol. (ul)	0	Plate Pos.		IRM Status			
Data File	POPB.D	Method (Acq)	HE-HC.M	Comment		Acq. Time (Local)	2023-05-23 5:44:31 PM (UTC-04:00)

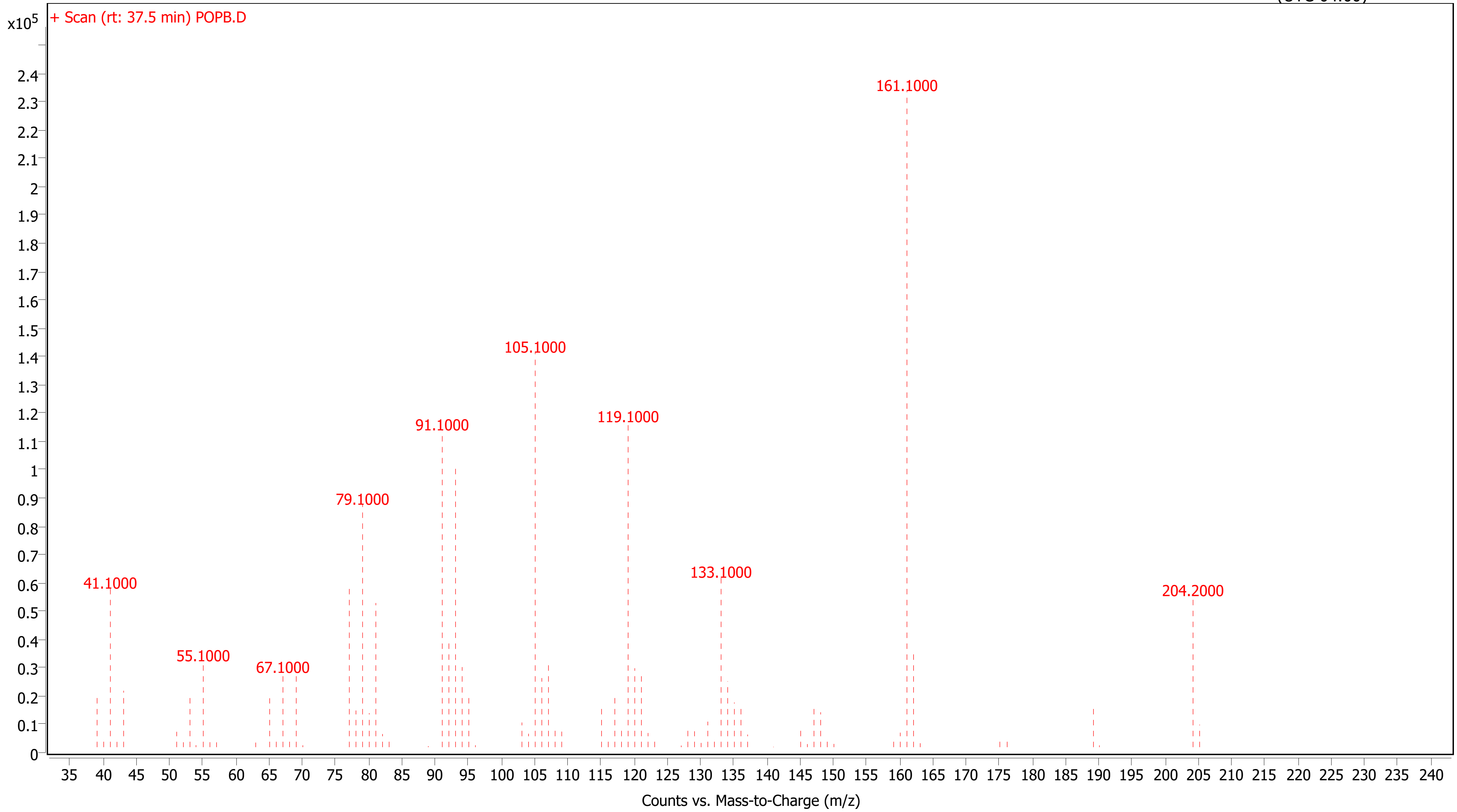

Spectrum Plot Report

Name	PopB	Rack Pos.		Instrument	GCMSD	Operator	Heloise
Inj. Vol. (ul)	0	Plate Pos.		IRM Status			
Data File	POPB.D	Method (Acq)	HE-HC.M	Comment		Acq. Time (Local)	2023-05-23 5:44:31 PM (UTC-04:00)

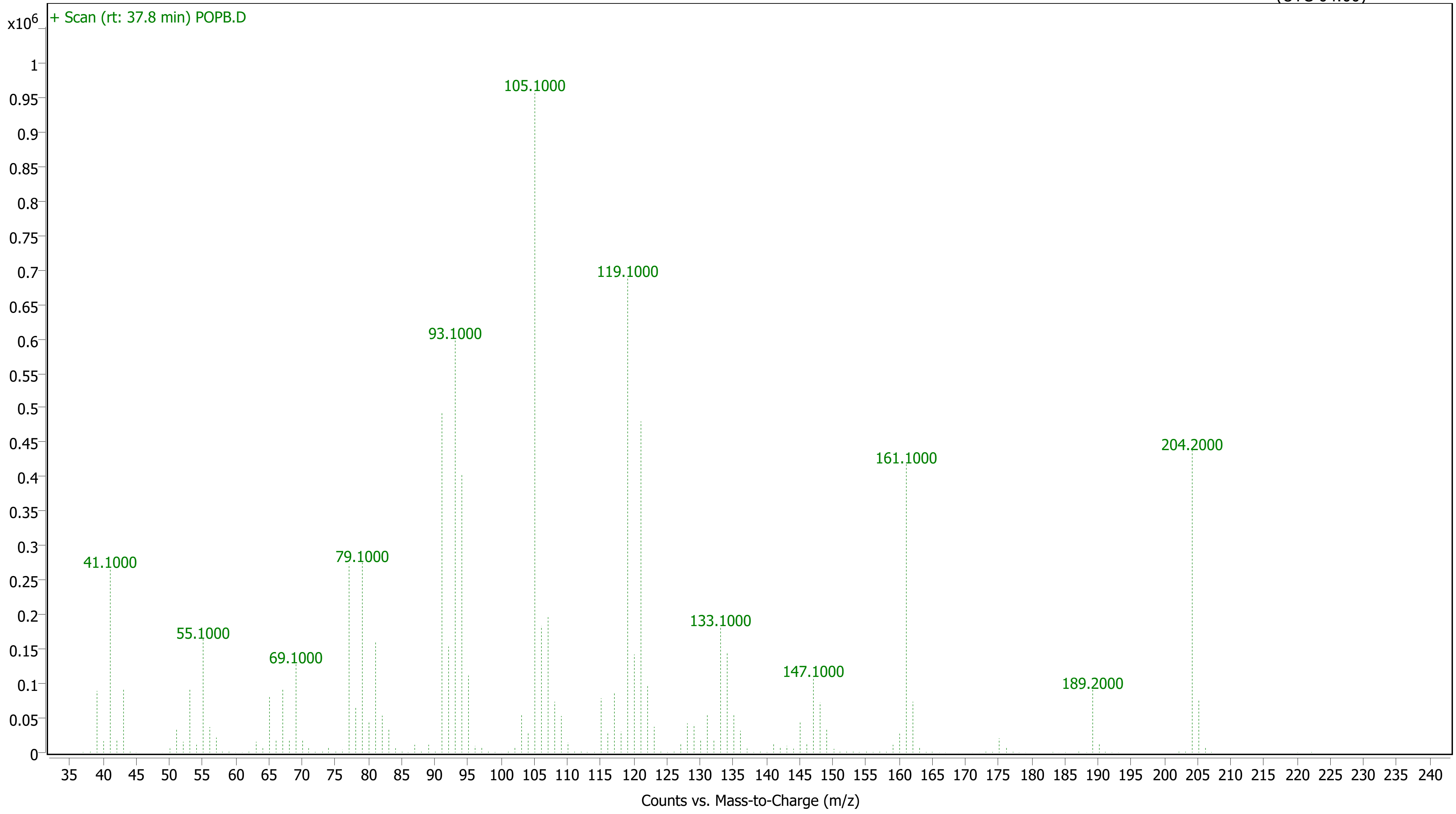

Spectrum Plot Report

Name	PopB	Rack Pos.		Instrument	GCMSD	Operator	Heloise
Inj. Vol. (ul)	0	Plate Pos.		IRM Status			
Data File	POPB.D	Method (Acq)	HE-HC.M	Comment		Acq. Time (Local)	2023-05-23 5:44:31 PM (UTC-04:00)

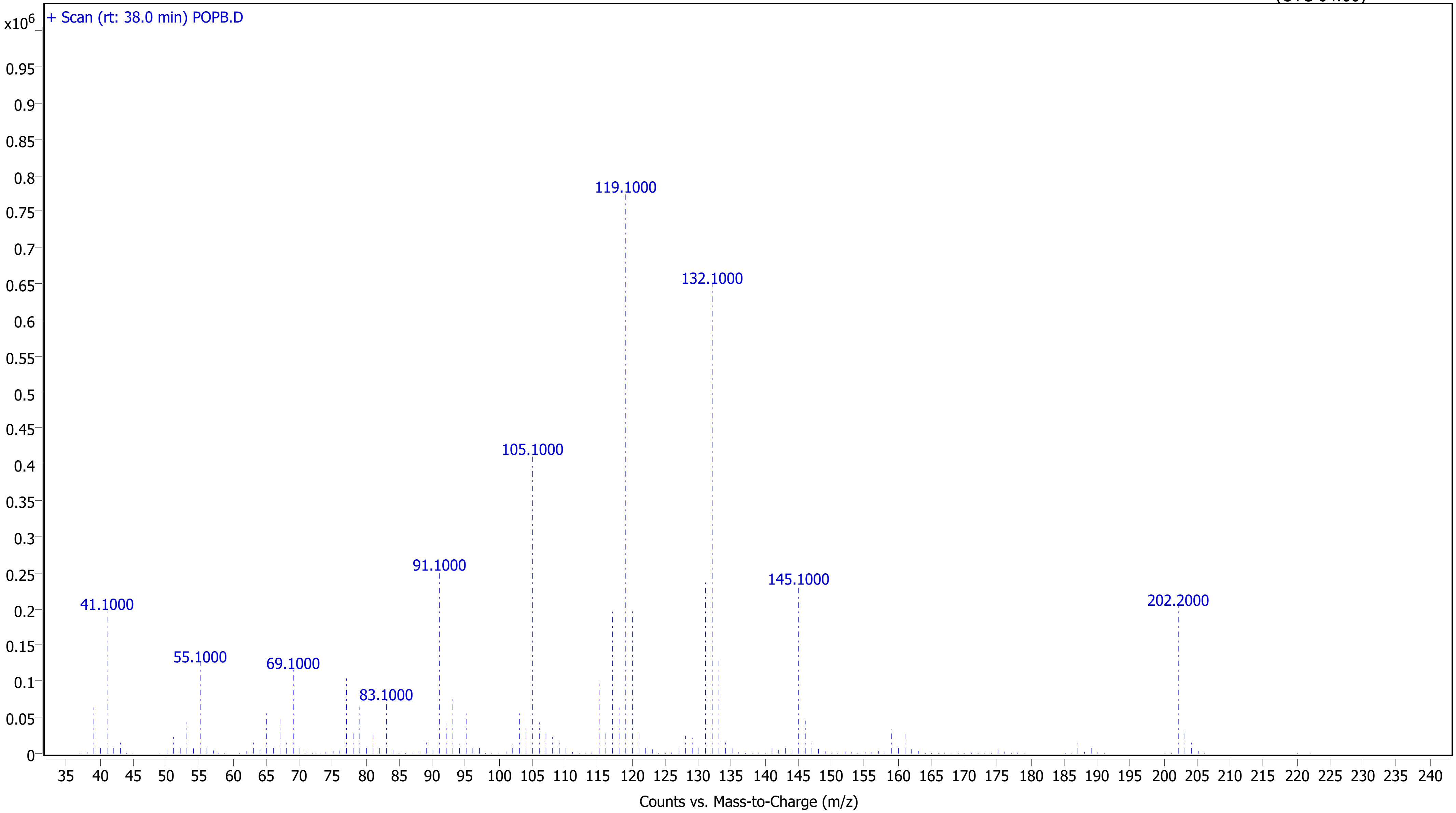

Spectrum Plot Report

Agilent Trusted Answers

Name	PopB	Rack Pos.		Instrument	GCMSD	Operator	Heloise
Inj. Vol. (ul)	0	Plate Pos.		IRM Status			
Data File	POPB.D	Method (Acq)	HE-HC.M	Comment		Acq. Time (Local)	2023-05-23 5:44:31 PM (UTC-04:00)

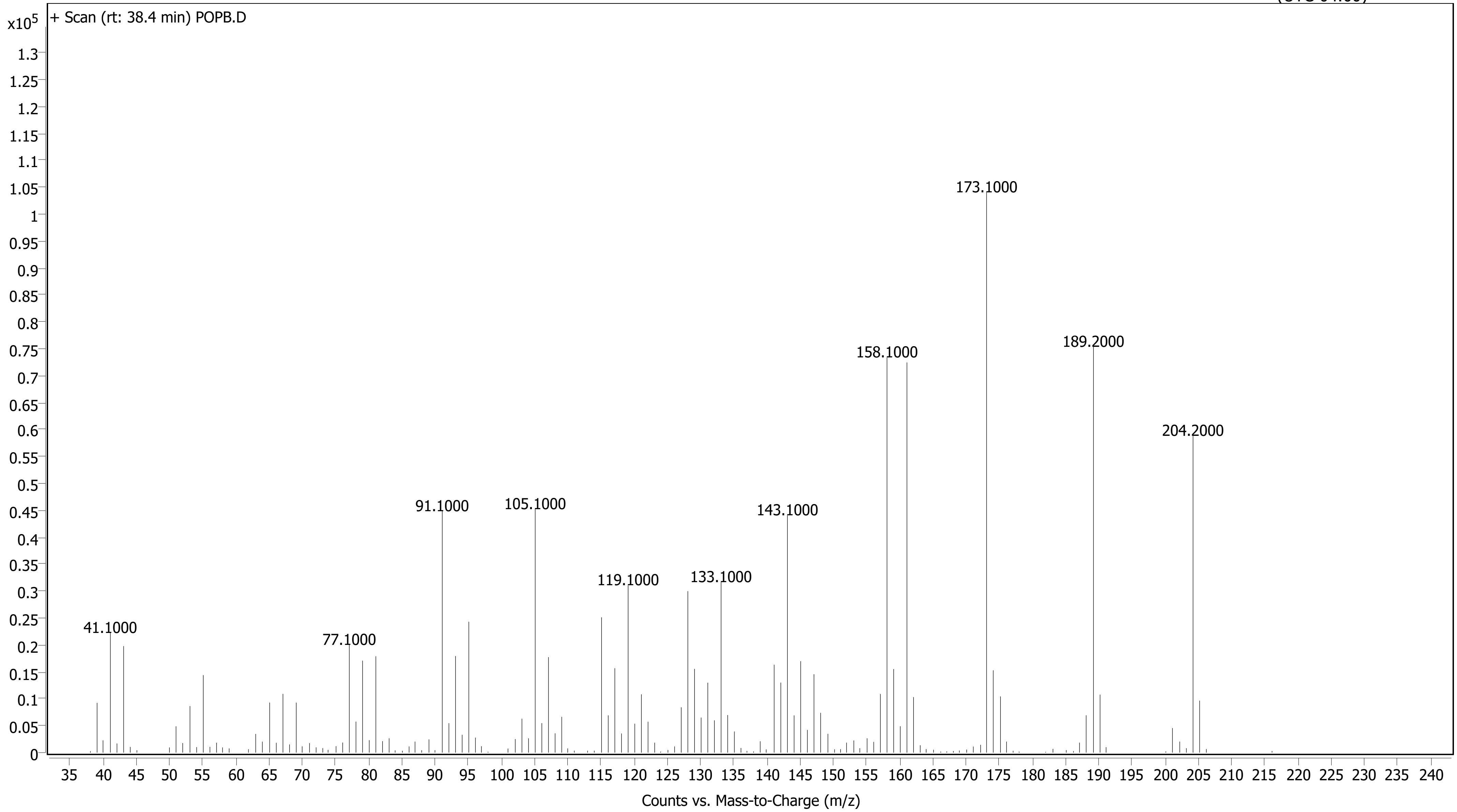

Spectrum Plot Report

Name	PopB	Rack Pos.		Instrument	GCMSD	Operator	Heloise
Inj. Vol. (ul)	0	Plate Pos.		IRM Status			
Data File	POPB.D	Method (Acq)	HE-HC.M	Comment		Acq. Time (Local)	2023-05-23 5:44:31 PM (UTC-04:00)

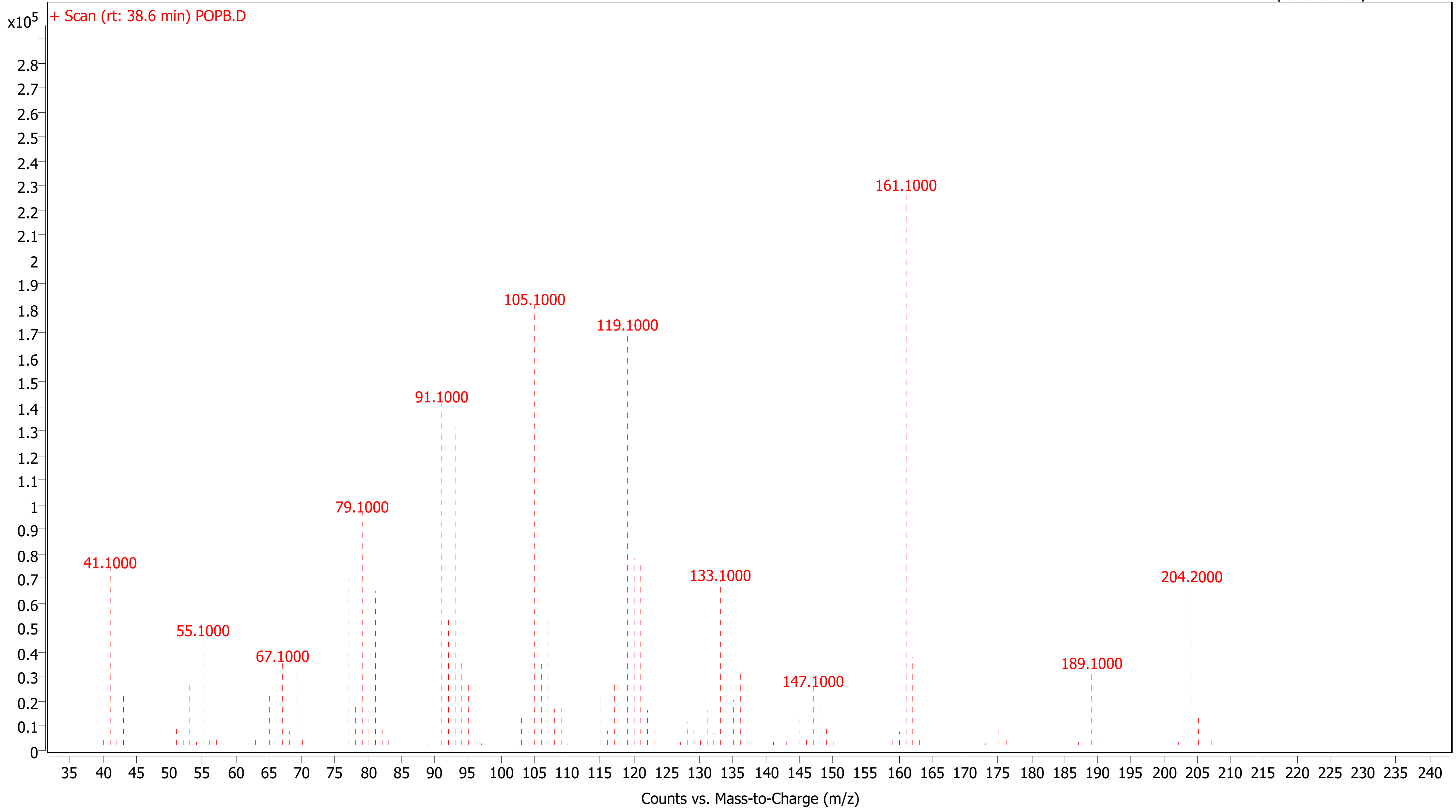

Generated at 6:02 PM on 2026-04-17

Spectrum Plot Report

Name	PopB	Rack Pos.		Instrument	GCMSD	Operator	Heloise
Inj. Vol. (ul)	0	Plate Pos.		IRM Status			
Data File	POPB.D	Method (Acq)	HE-HC.M	Comment		Acq. Time (Local)	2023-05-23 5:44:31 PM (UTC-04:00)

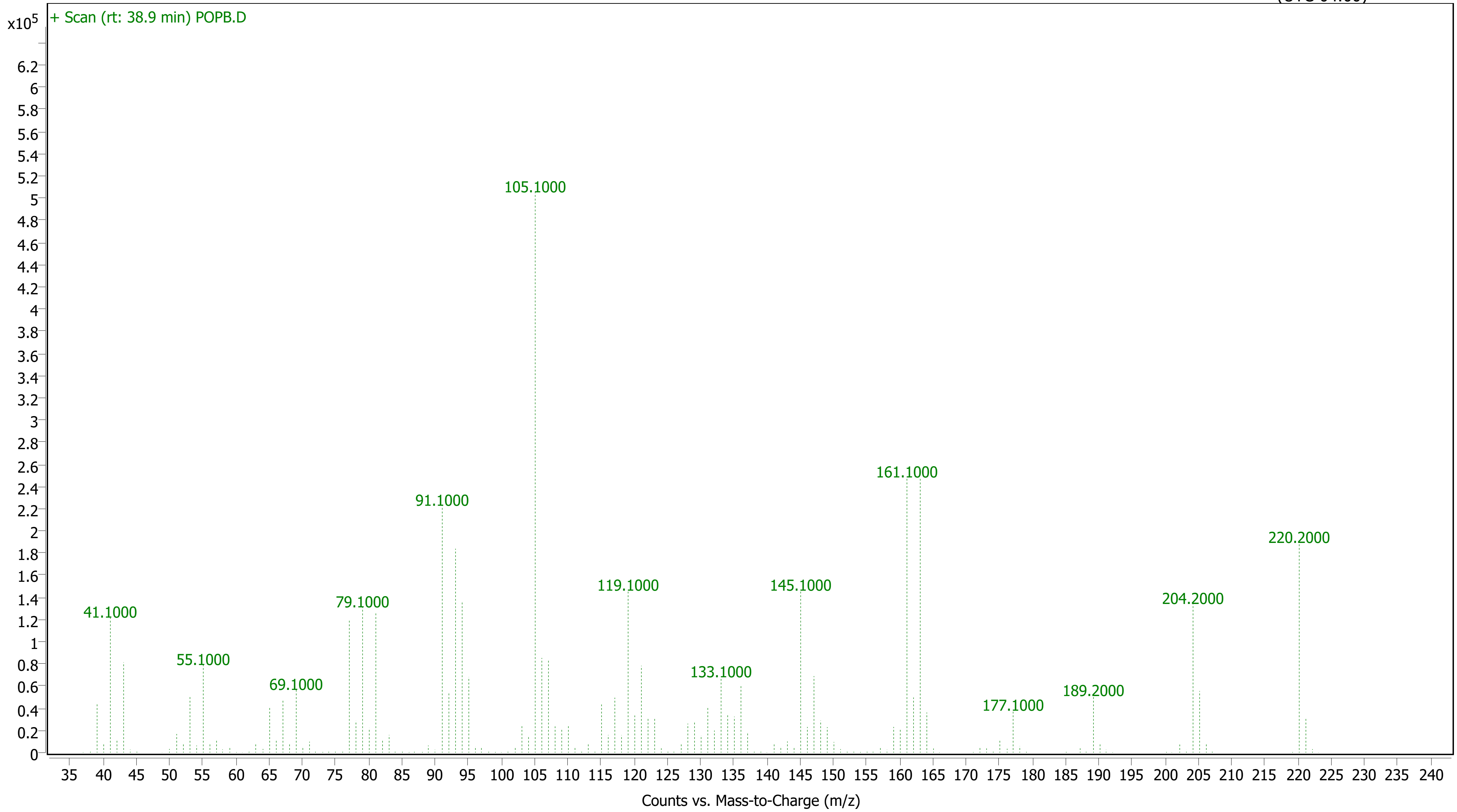

Generated at 6:02 PM on 2026-04-17

Spectrum Plot Report

Name	PopB	Rack Pos.		Instrument	GCMSD	Operator	Heloise
Inj. Vol. (ul)	0	Plate Pos.		IRM Status			
Data File	POPB.D	Method (Acq)	HE-HC.M	Comment		Acq. Time (Local)	2023-05-23 5:44:31 PM (UTC-04:00)

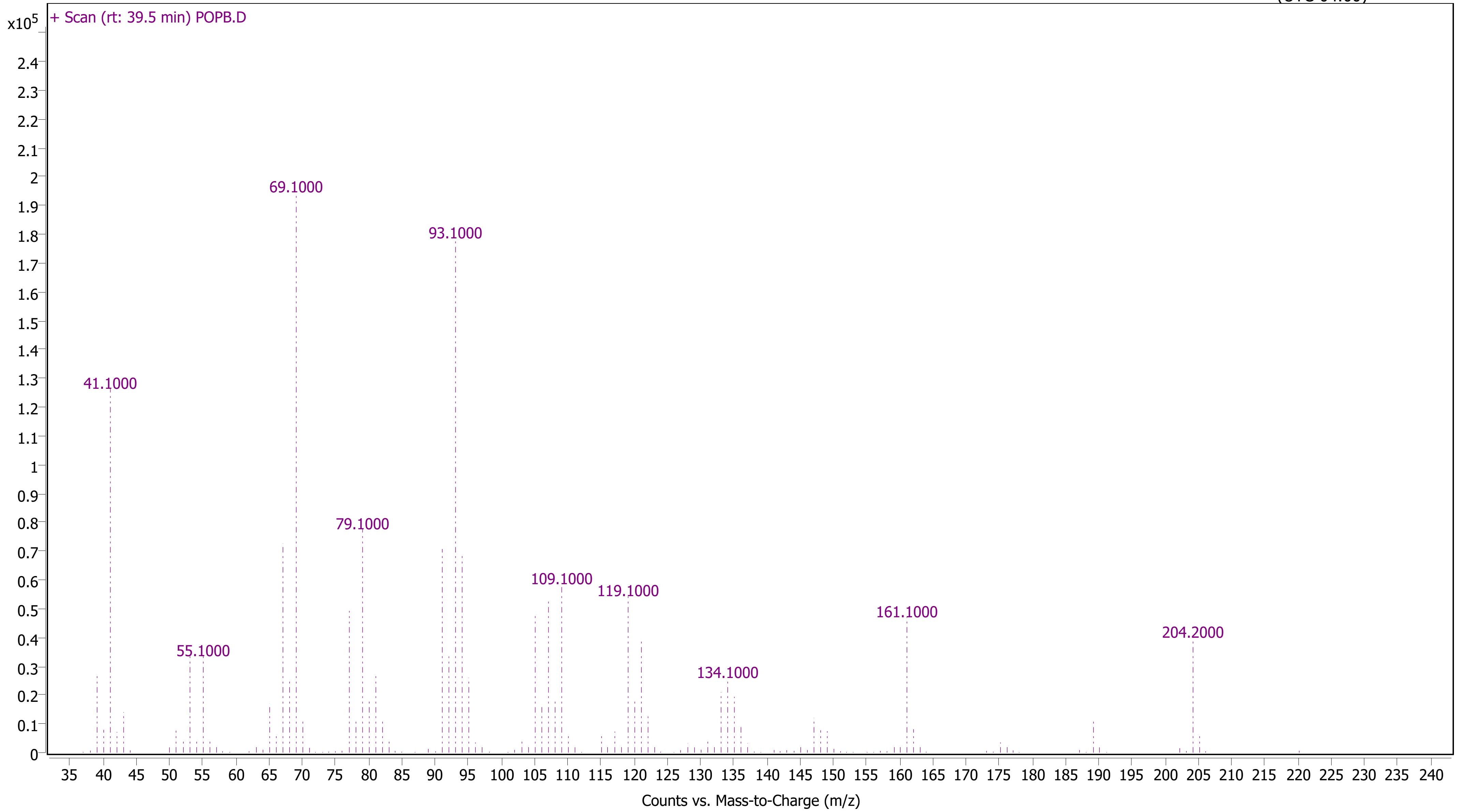

Spectrum Plot Report

Name	PopB	Rack Pos.		Instrument	GCMSD	Operator	Heloise
Inj. Vol. (ul)	0	Plate Pos.		IRM Status			
Data File	POPB.D	Method (Acq)	HE-HC.M	Comment		Acq. Time (Local)	2023-05-23 5:44:31 PM (UTC-04:00)

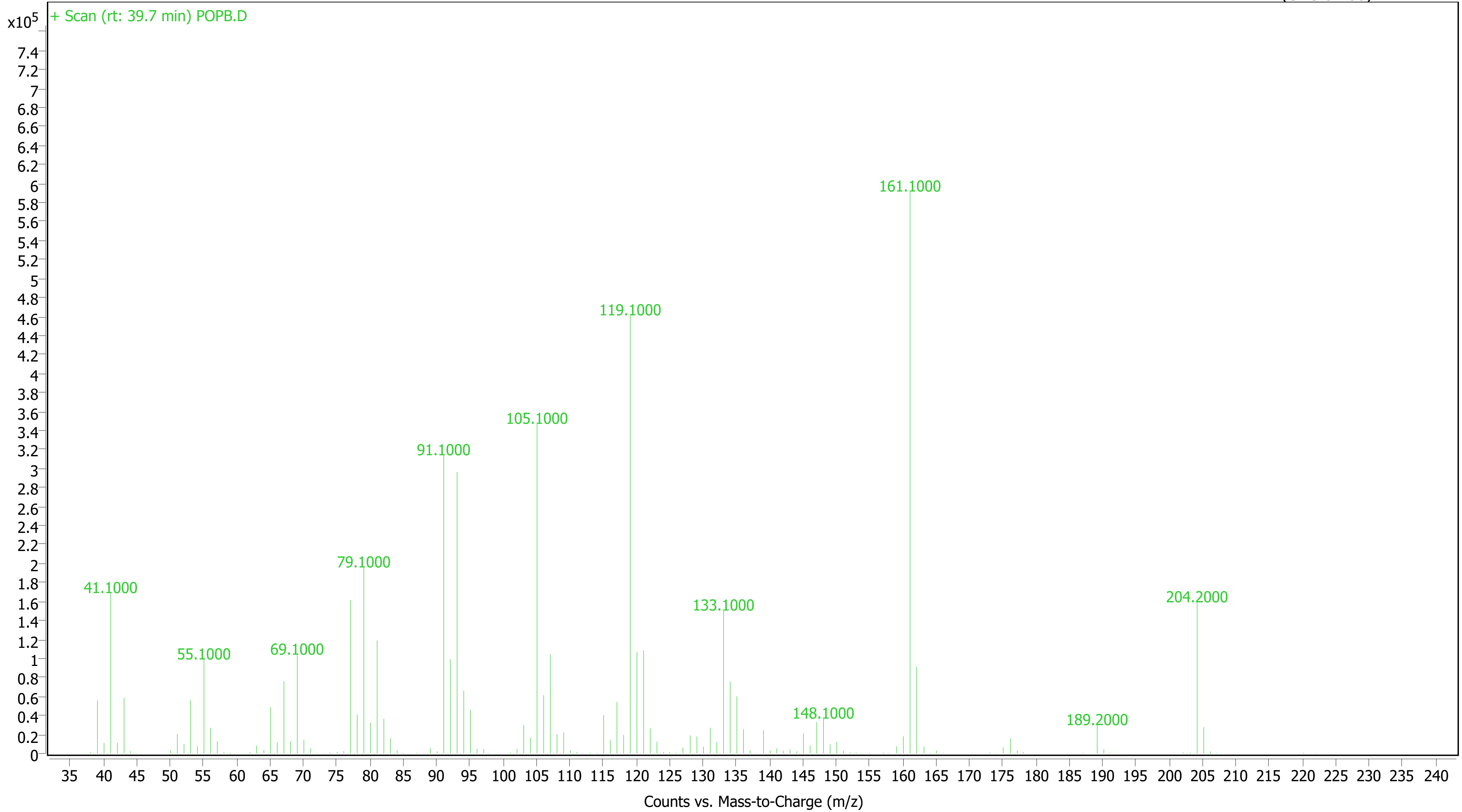

Spectrum Plot Report

Name	PopB	Rack Pos.		Instrument	GCMSD	Operator	Heloise
Inj. Vol. (ul)	0	Plate Pos.		IRM Status			
Data File	POPB.D	Method (Acq)	HE-HC.M	Comment		Acq. Time (Local)	2023-05-23 5:44:31 PM (UTC-04:00)

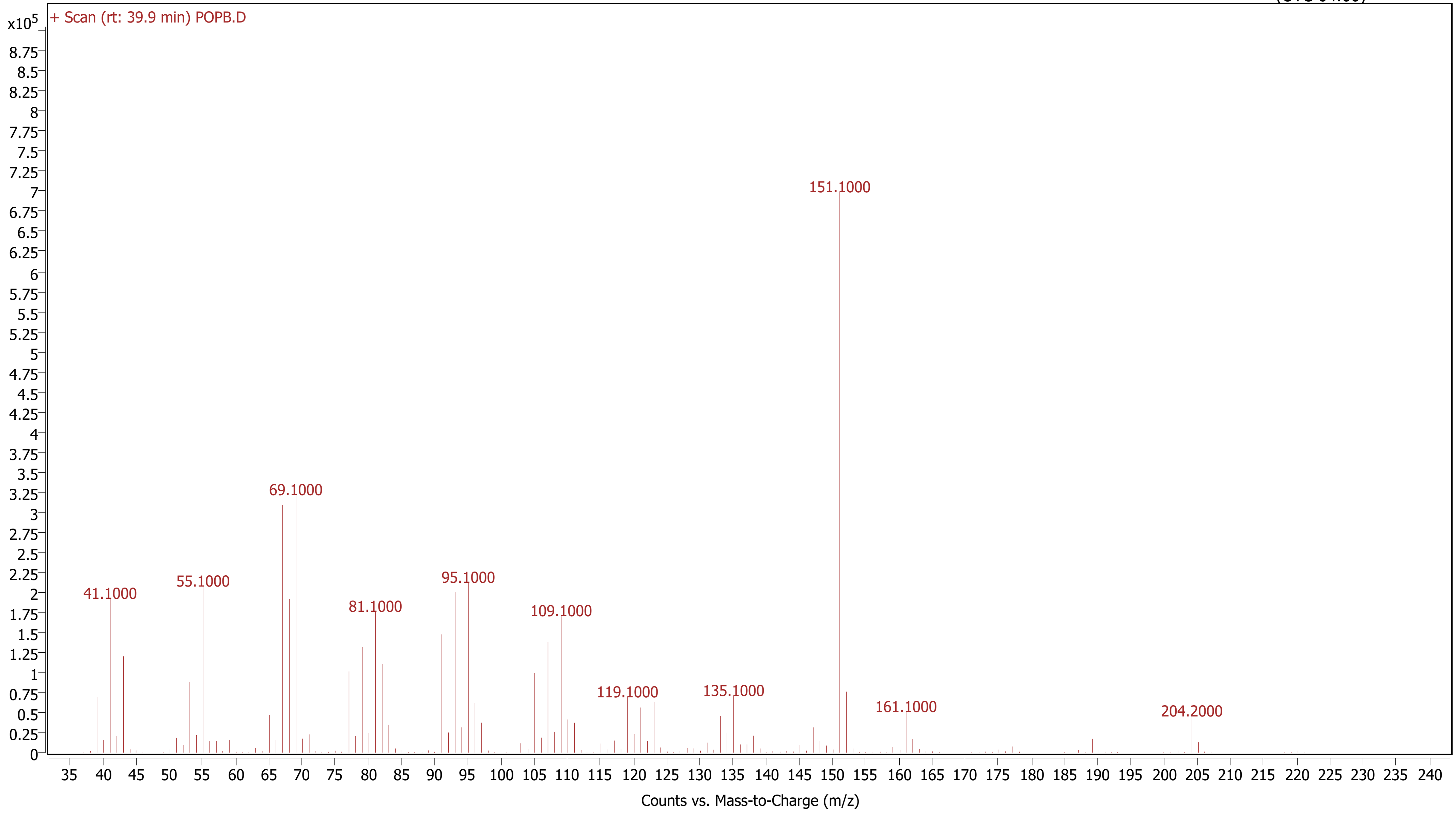

Generated at 6:02 PM on 2026-04-17

Spectrum Plot Report

Name	PopB	Rack Pos.		Instrument	GCMSD	Operator	Heloise
Inj. Vol. (ul)	0	Plate Pos.		IRM Status			
Data File	POPB.D	Method (Acq)	HE-HC.M	Comment		Acq. Time (Local)	2023-05-23 5:44:31 PM (UTC-04:00)

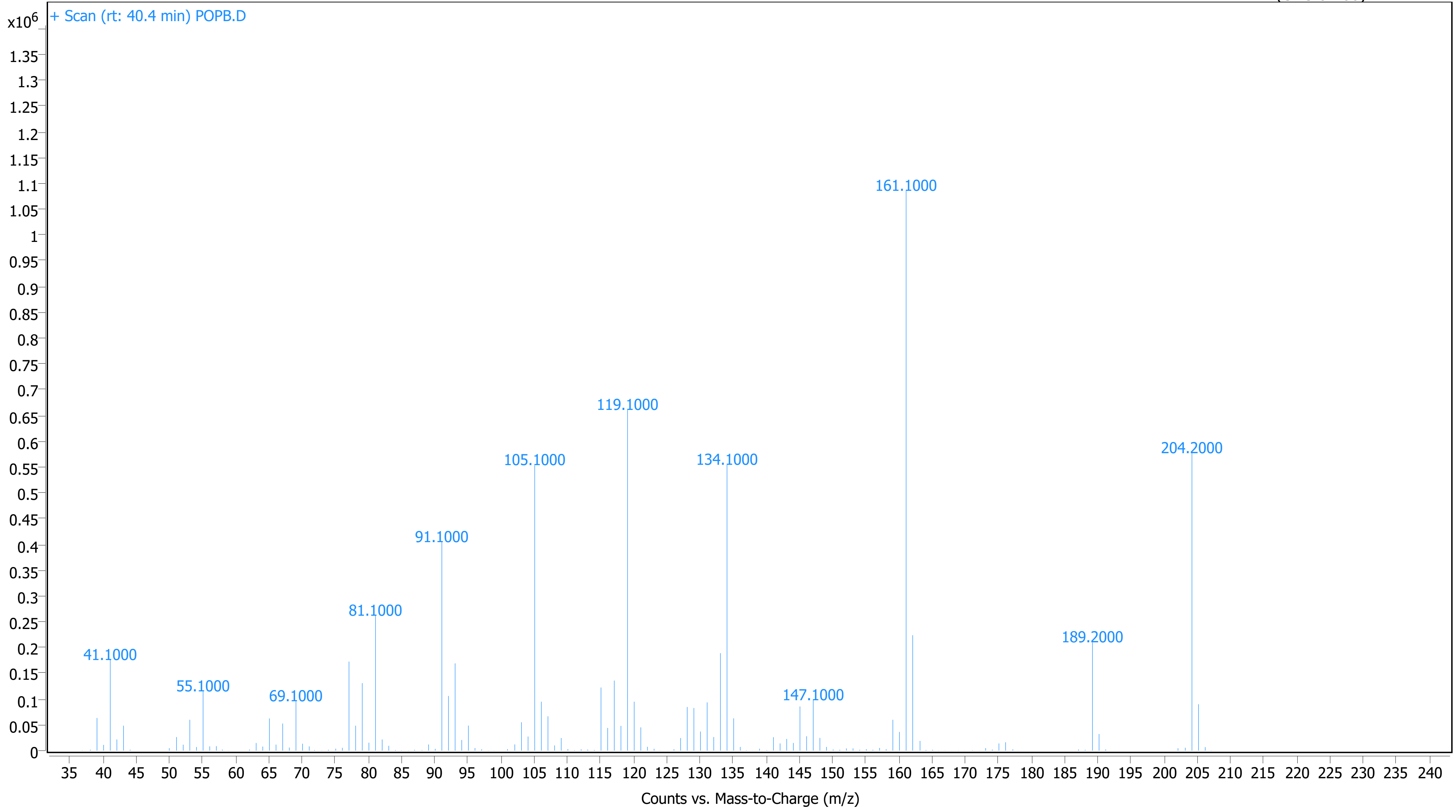

Spectrum Plot Report

Name	PopB	Rack Pos.		Instrument	GCMSD	Operator	Heloise
Inj. Vol. (ul)	0	Plate Pos.		IRM Status			
Data File	POPB.D	Method (Acq)	HE-HC.M	Comment		Acq. Time (Local)	2023-05-23 5:44:31 PM (UTC-04:00)

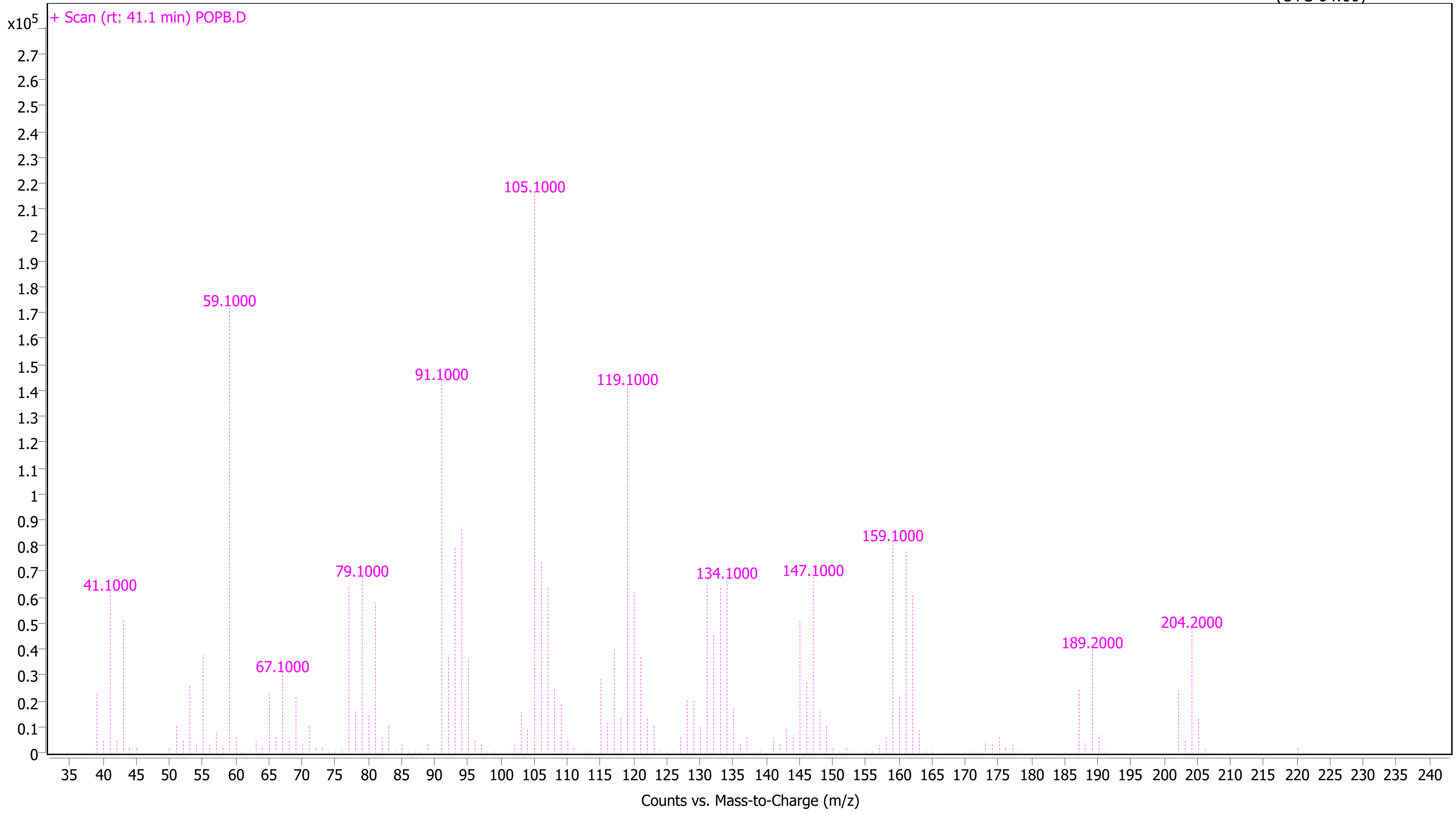

Spectrum Plot Report

Name	PopB	Rack Pos.		Instrument	GCMSD	Operator	Heloise
Inj. Vol. (ul)	0	Plate Pos.		IRM Status			
Data File	POPB.D	Method (Acq)	HE-HC.M	Comment		Acq. Time (Local)	2023-05-23 5:44:31 PM (UTC-04:00)

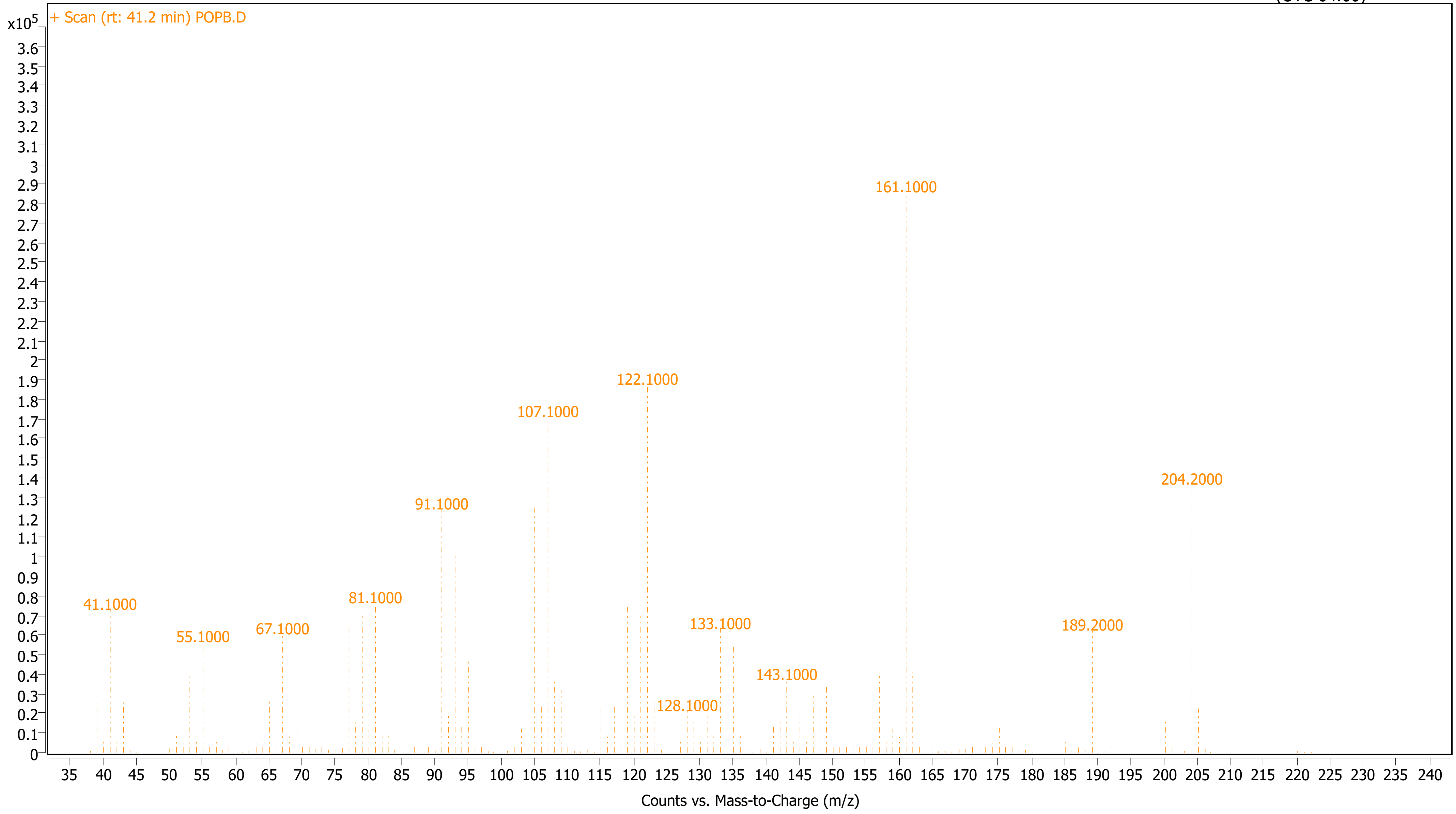

Generated at 6:02 PM on 2026-04-17

Spectrum Plot Report

Name	PopB	Rack Pos.		Instrument	GCMSD	Operator	Heloise
Inj. Vol. (ul)	0	Plate Pos.		IRM Status			
Data File	POPB.D	Method (Acq)	HE-HC.M	Comment		Acq. Time (Local)	2023-05-23 5:44:31 PM (UTC-04:00)

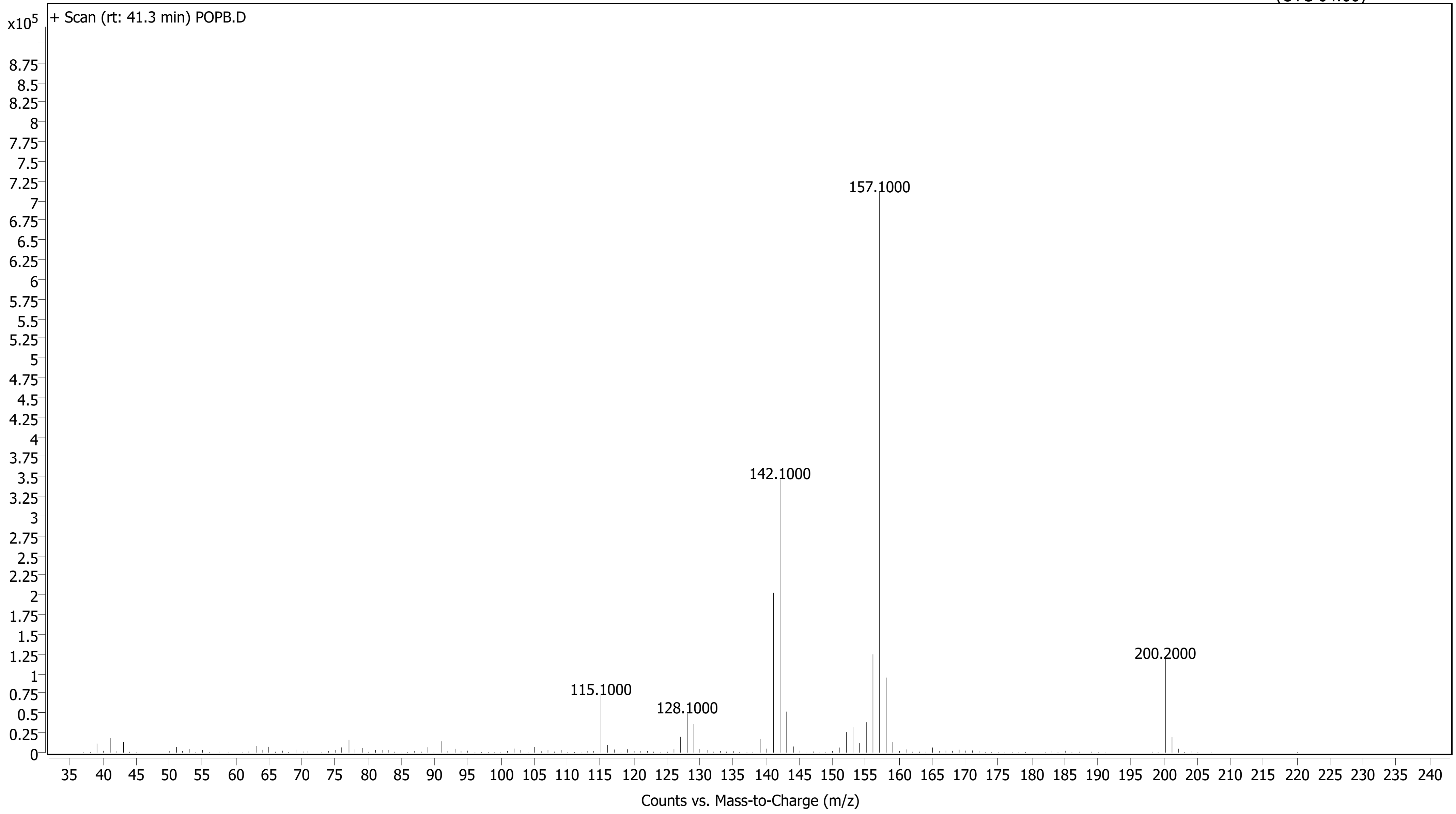

Spectrum Plot Report

Name	PopB	Rack Pos.		Instrument	GCMSD	Operator	Heloise
Inj. Vol. (ul)	0	Plate Pos.		IRM Status			
Data File	POPB.D	Method (Acq)	HE-HC.M	Comment		Acq. Time (Local)	2023-05-23 5:44:31 PM (UTC-04:00)

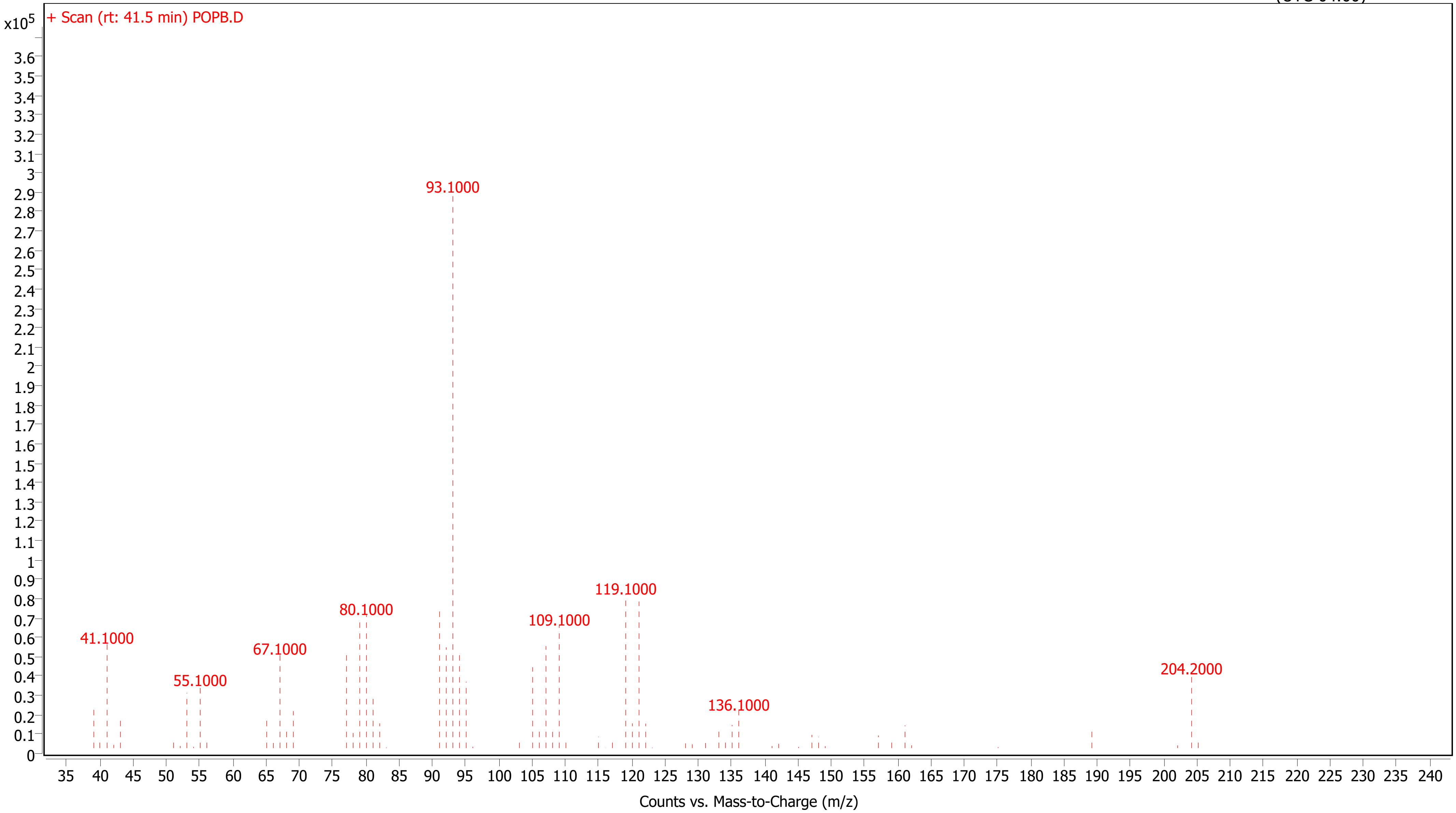

Spectrum Plot Report

Name	PopB	Rack Pos.		Instrument	GCMSD	Operator	Heloise
Inj. Vol. (ul)	0	Plate Pos.		IRM Status			
Data File	POPB.D	Method (Acq)	HE-HC.M	Comment		Acq. Time (Local)	2023-05-23 5:44:31 PM (UTC-04:00)

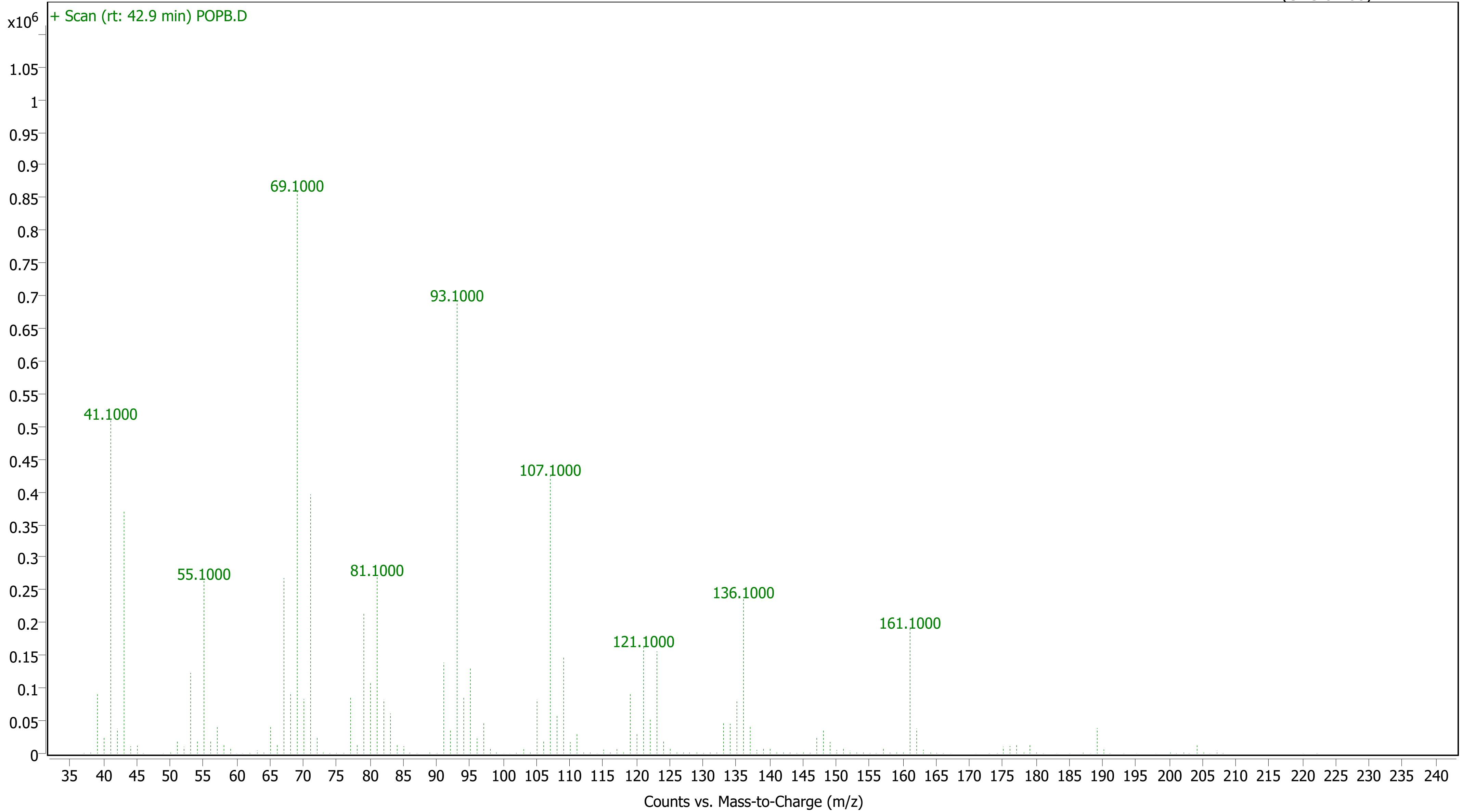

Spectrum Plot Report

Name	PopB	Rack Pos.		Instrument	GCMSD	Operator	Heloise
Inj. Vol. (ul)	0	Plate Pos.		IRM Status			
Data File	POPB.D	Method (Acq)	HE-HC.M	Comment		Acq. Time (Local)	2023-05-23 5:44:31 PM (UTC-04:00)

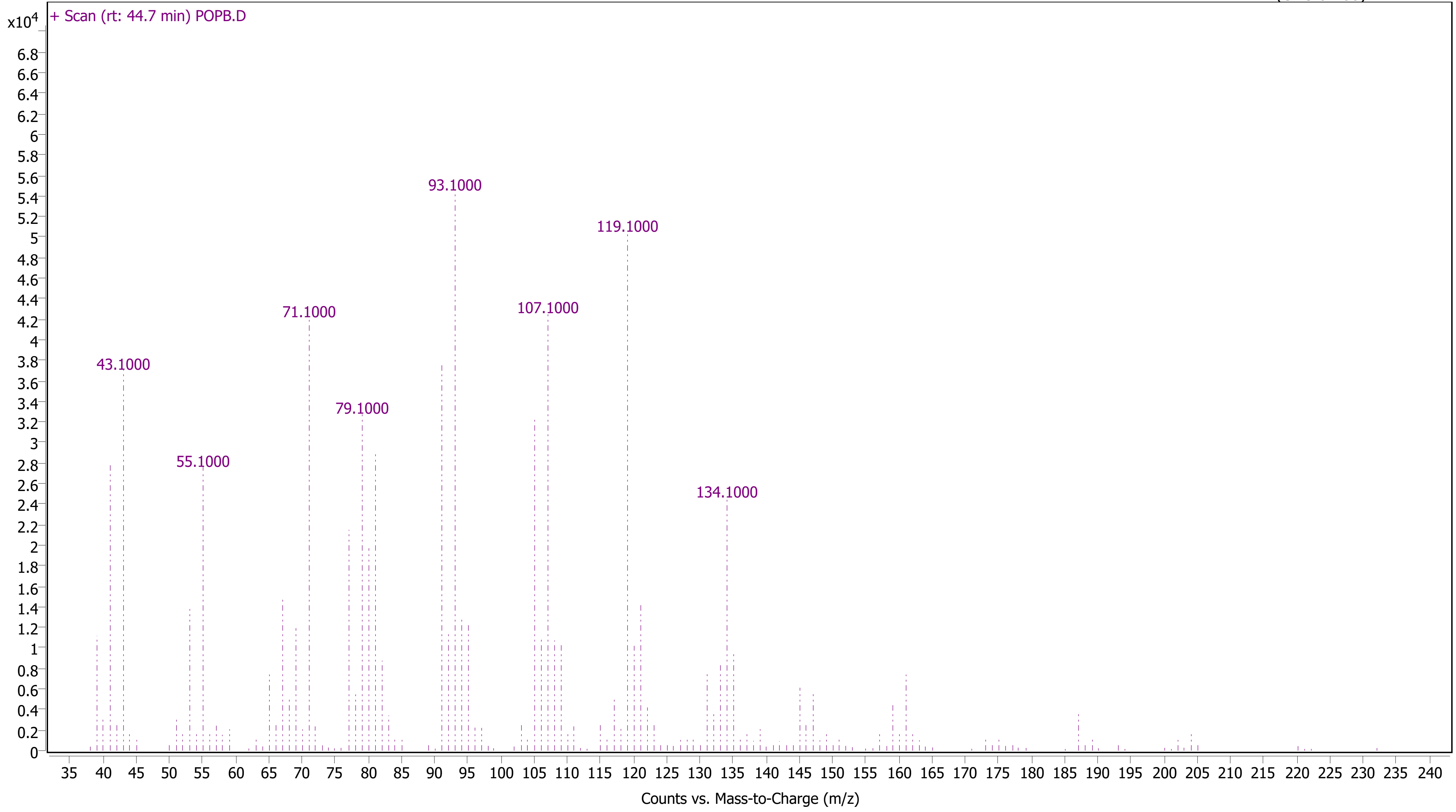

Generated at 6:02 PM on 2026-04-17

Spectrum Plot Report

Name	PopB	Rack Pos.		Instrument	GCMSD	Operator	Heloise
Inj. Vol. (ul)	0	Plate Pos.		IRM Status			
Data File	POPB.D	Method (Acq)	HE-HC.M	Comment		Acq. Time (Local)	2023-05-23 5:44:31 PM (UTC-04:00)

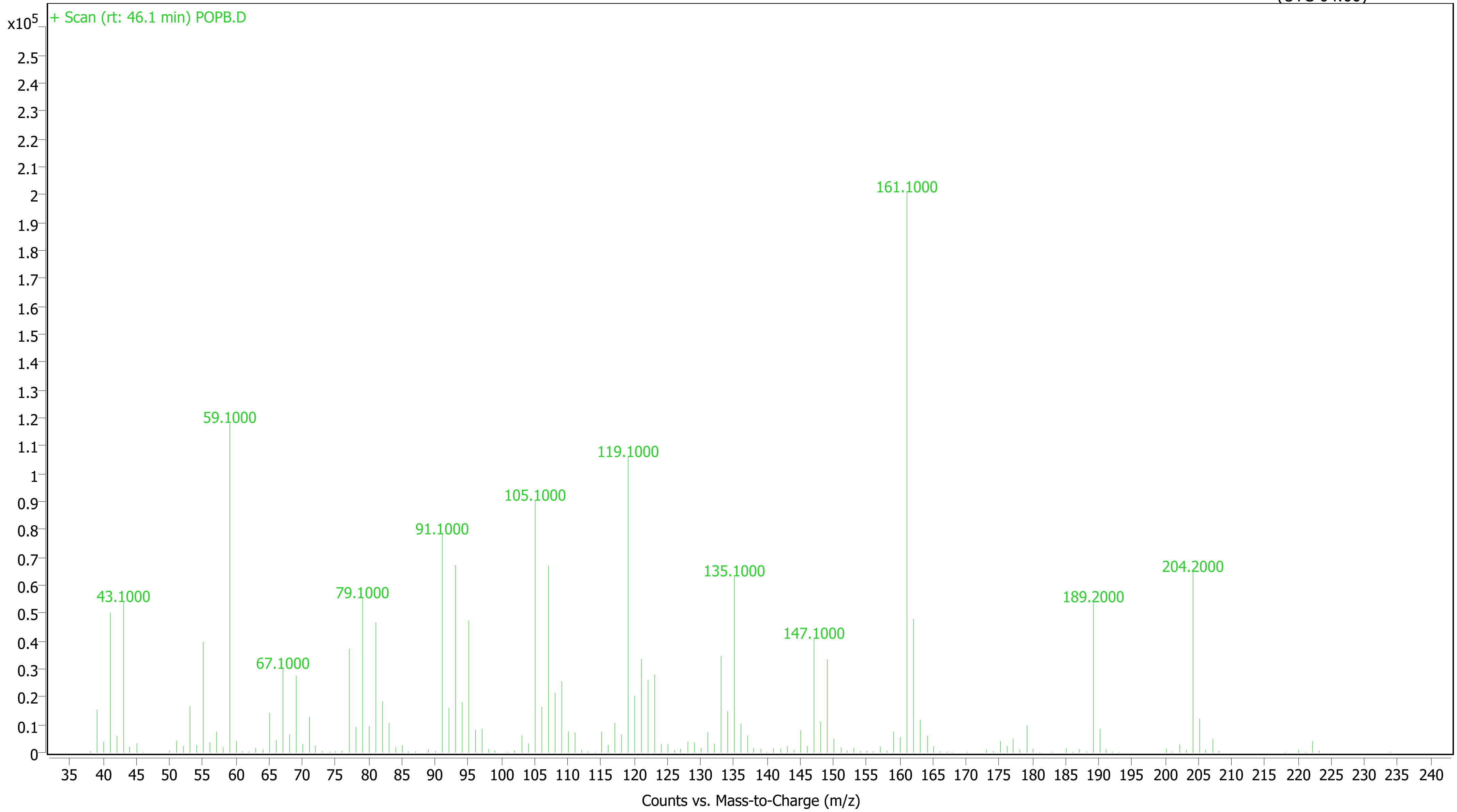

Spectrum Plot Report

Name	PopB	Rack Pos.		Instrument	GCMSD	Operator	Heloise
Inj. Vol. (ul)	0	Plate Pos.		IRM Status			
Data File	POPB.D	Method (Acq)	HE-HC.M	Comment		Acq. Time (Local)	2023-05-23 5:44:31 PM (UTC-04:00)

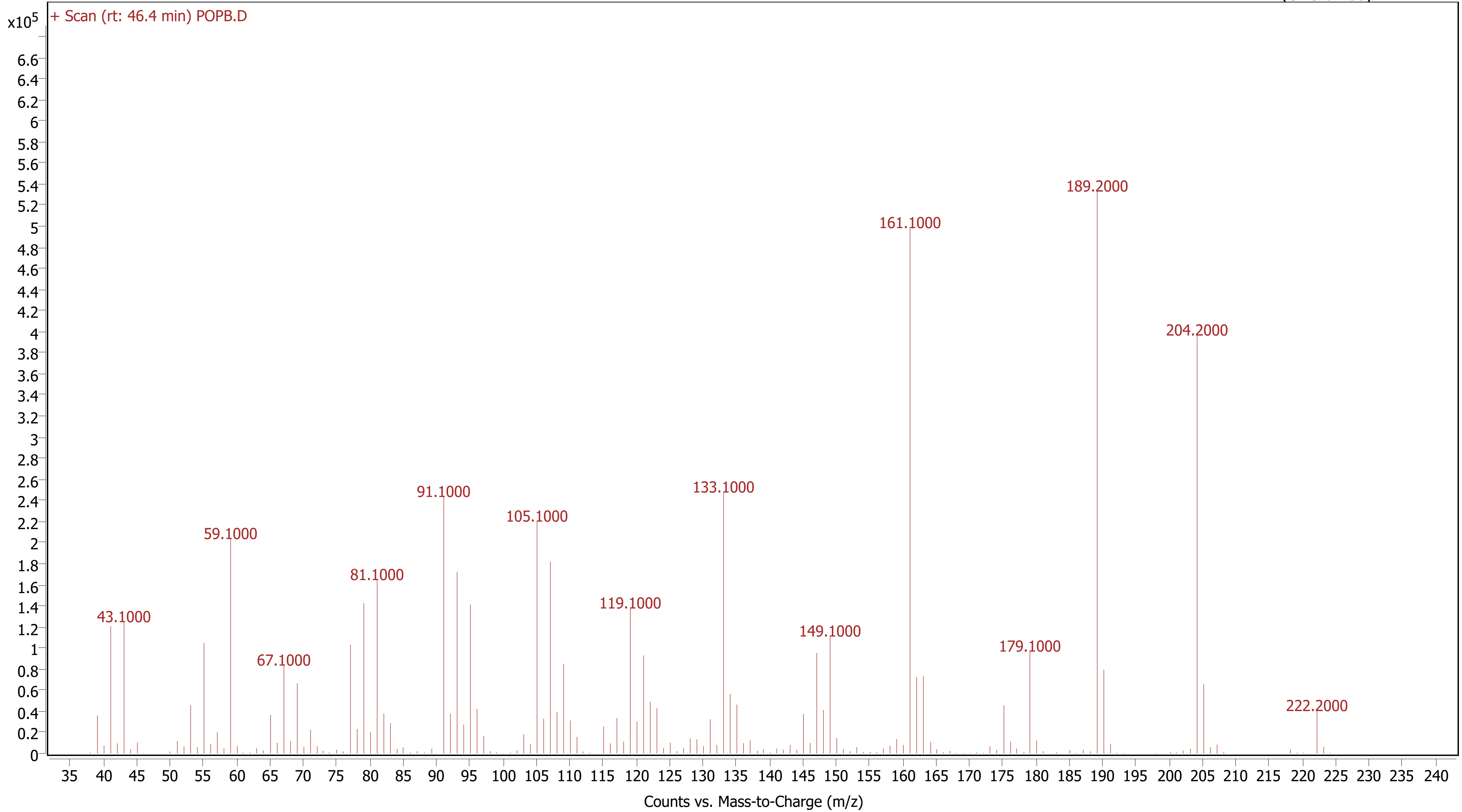

Spectrum Plot Report

Name	PopB	Rack Pos.		Instrument	GCMSD	Operator	Heloise
Inj. Vol. (ul)	0	Plate Pos.		IRM Status			
Data File	POPB.D	Method (Acq)	HE-HC.M	Comment		Acq. Time (Local)	2023-05-23 5:44:31 PM (UTC-04:00)

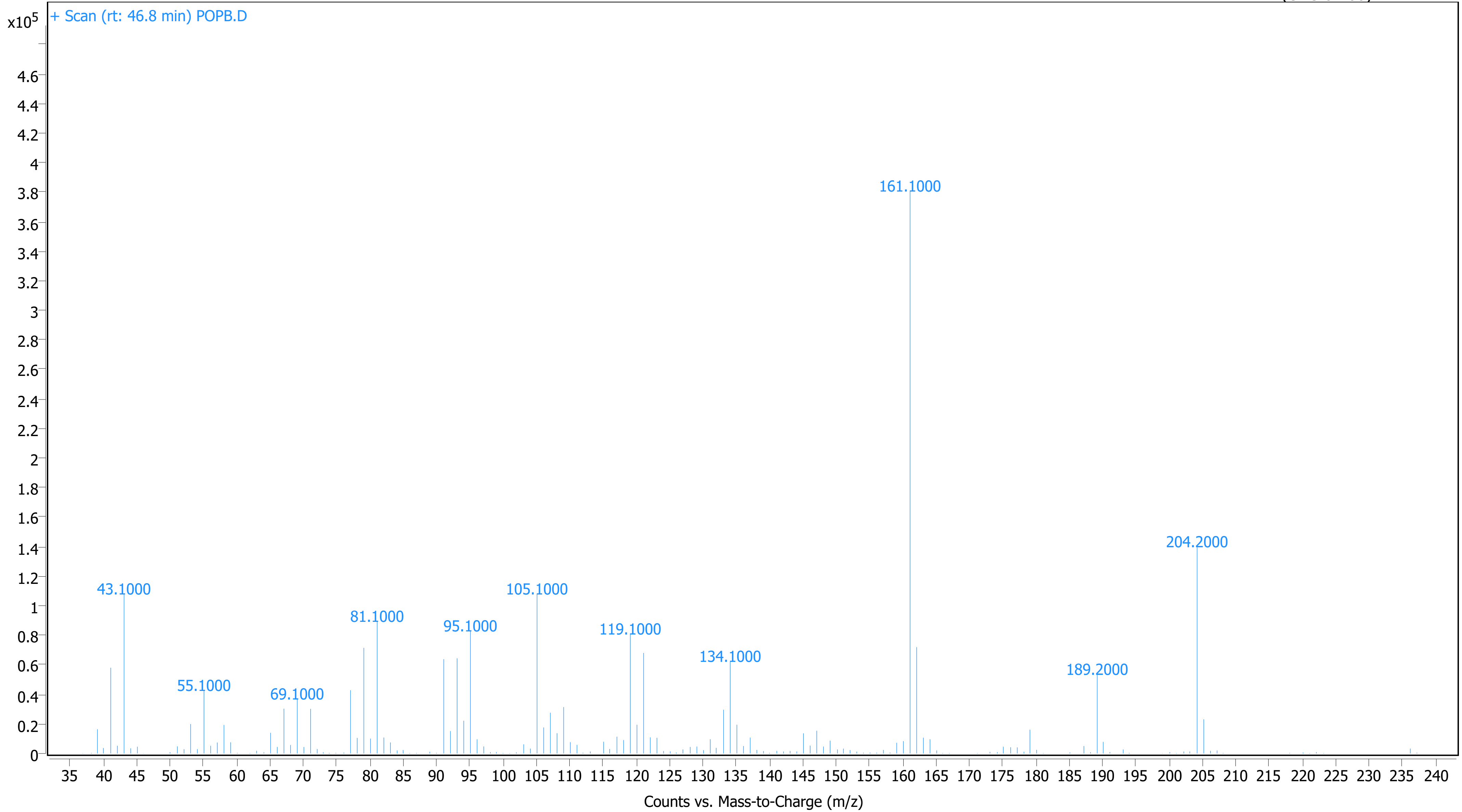

Spectrum Plot Report

Agilent Trusted Answers

Name	PopB	Rack Pos.		Instrument	GCMSD	Operator	Heloise
Inj. Vol. (ul)	0	Plate Pos.		IRM Status			
Data File	POPB.D	Method (Acq)	HE-HC.M	Comment		Acq. Time (Local)	2023-05-23 5:44:31 PM (UTC-04:00)

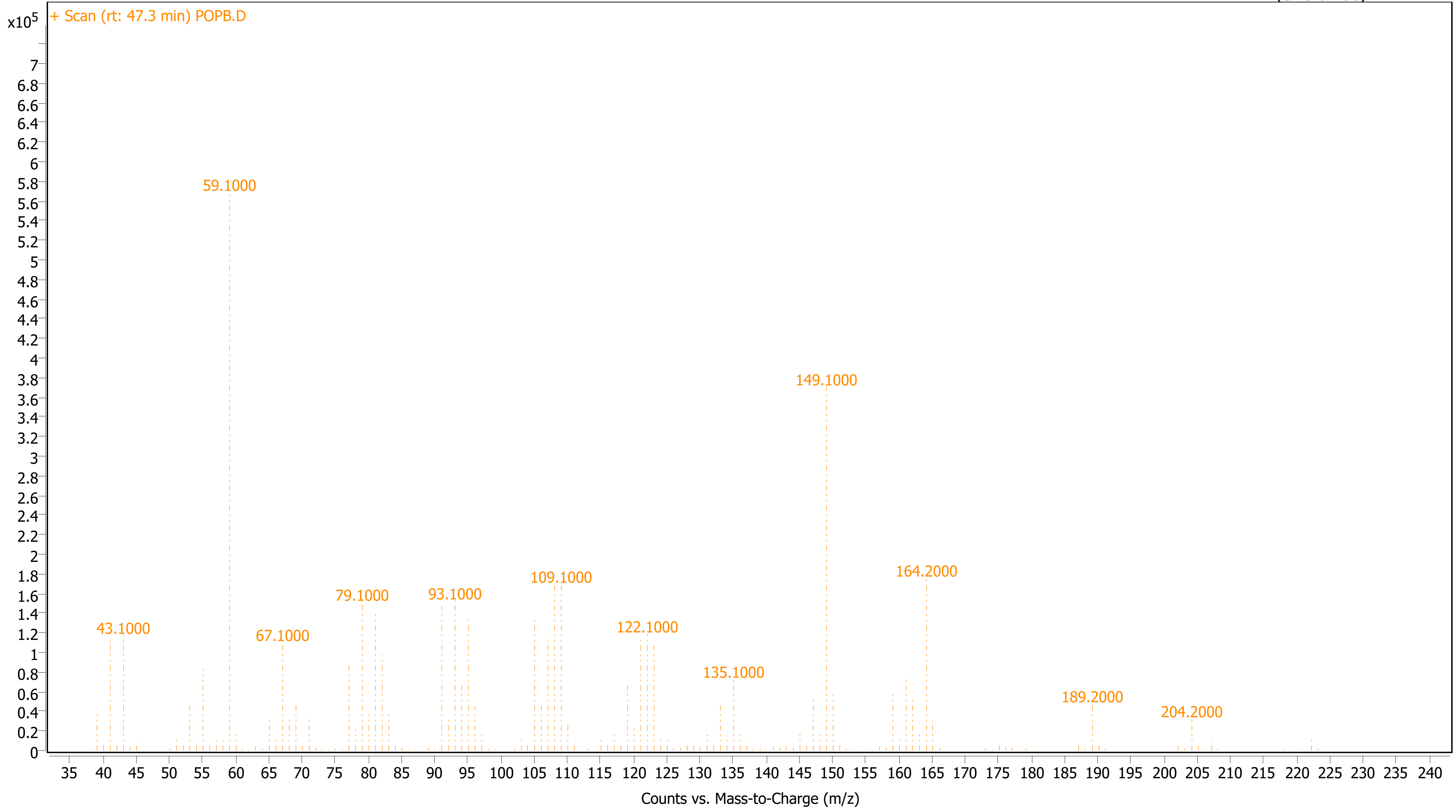

Generated at 6:02 PM on 2026-04-17

Spectrum Plot Report

Name	PopB	Rack Pos.		Instrument	GCMSD	Operator	Heloise
Inj. Vol. (ul)	0	Plate Pos.		IRM Status			
Data File	POPB.D	Method (Acq)	HE-HC.M	Comment		Acq. Time (Local)	2023-05-23 5:44:31 PM (UTC-04:00)

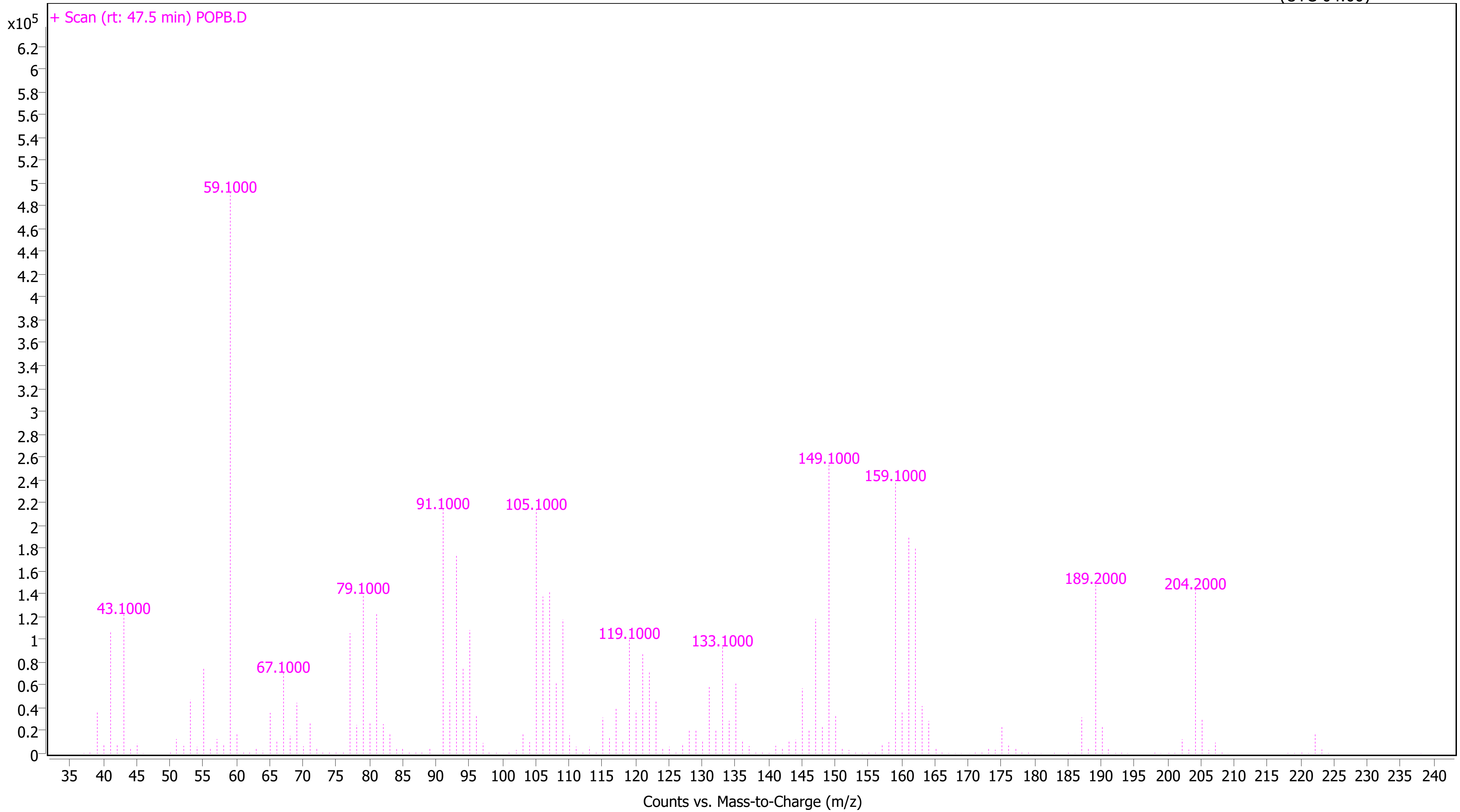

Spectrum Plot Report

Name	PopB	Rack Pos.		Instrument	GCMSD	Operator	Heloise
Inj. Vol. (ul)	0	Plate Pos.		IRM Status			
Data File	POPB.D	Method (Acq)	HE-HC.M	Comment		Acq. Time (Local)	2023-05-23 5:44:31 PM (UTC-04:00)

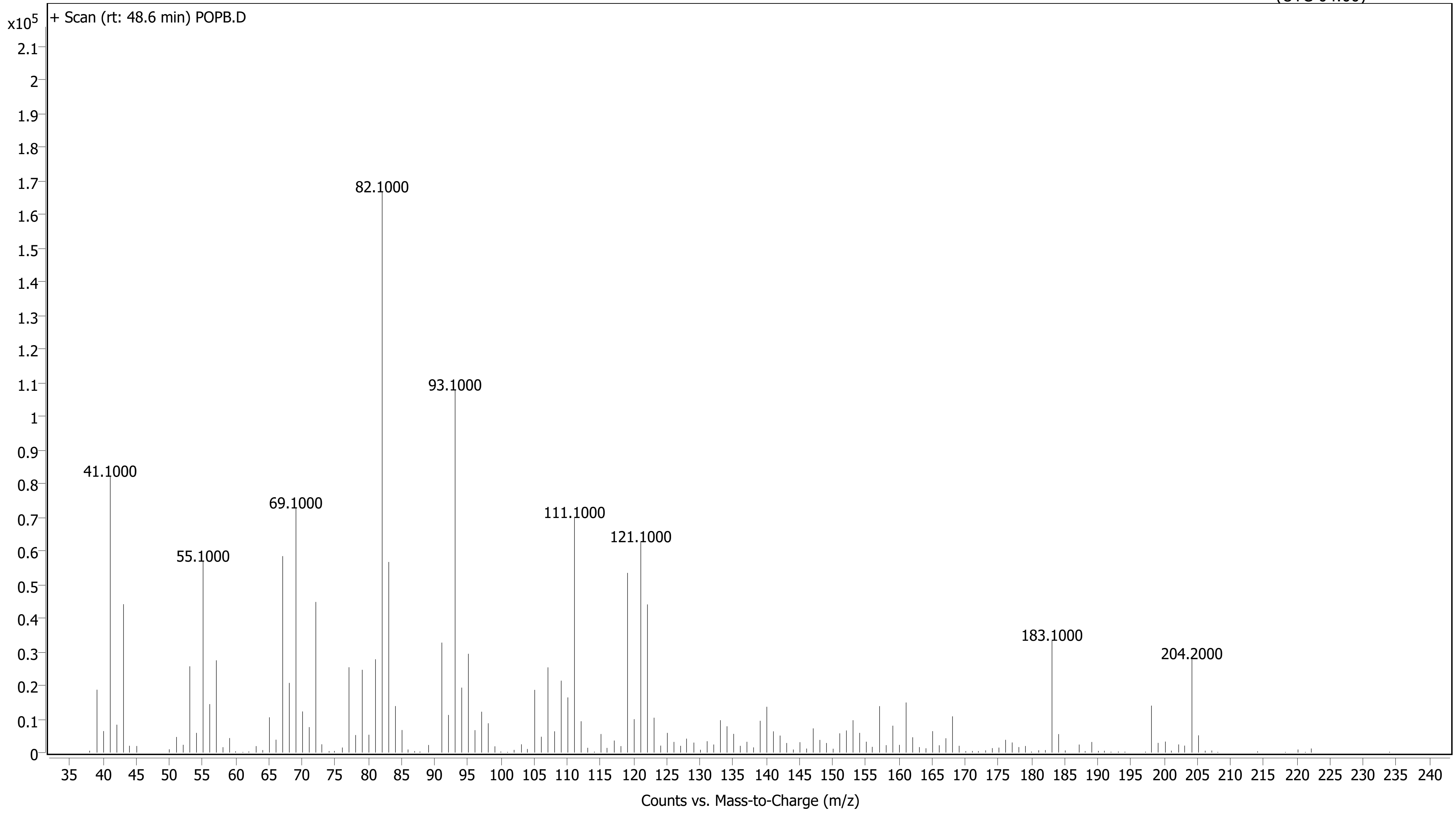

Spectrum Plot Report

Name	PopB	Rack Pos.		Instrument	GCMSD	Operator	Heloise
Inj. Vol. (ul)	0	Plate Pos.		IRM Status			
Data File	POPB.D	Method (Acq)	HE-HC.M	Comment		Acq. Time (Local)	2023-05-23 5:44:31 PM (UTC-04:00)

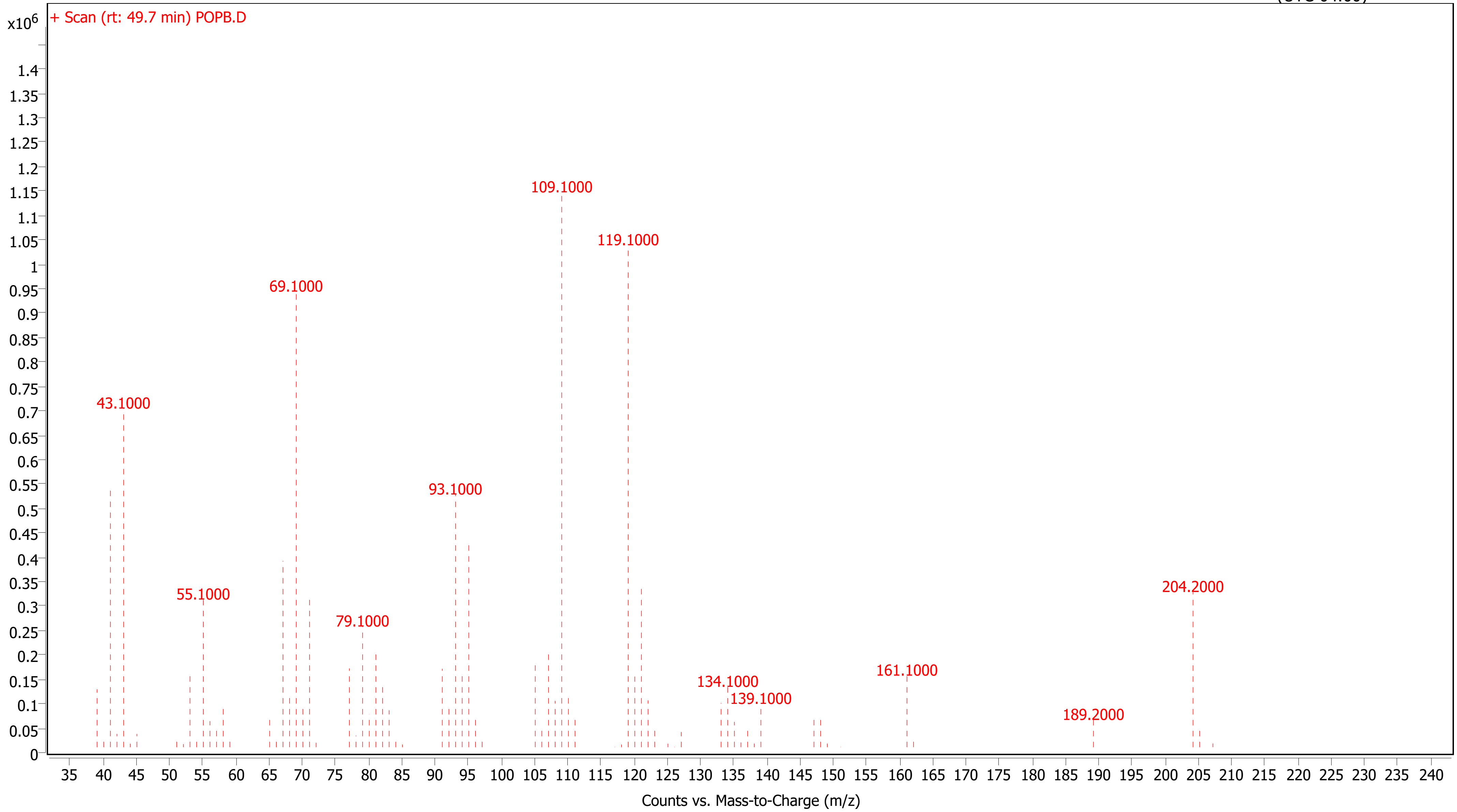

Supplement: Supplementary file 1 [file molecules-31-01496-s001.zip › molecules-4250645-supplementary.pdf]
